# Genome-wide diversity and admixture of five indigenous cattle populations from the Tigray region of northern Ethiopia

**DOI:** 10.3389/fgene.2023.1050365

**Published:** 2023-07-28

**Authors:** Tsadkan Zegeye, Gurja Belay, Adriana Vallejo-Trujillo, Jianlin Han, Olivier Hanotte

**Affiliations:** ^1^ Mekelle Agricultural Research Center, Tigray Agricultural Research Institute, Mekelle, Ethiopia; ^2^ Department of Microbial, Cellular and Molecular Biology, Addis Ababa University, Addis Ababa, Ethiopia; ^3^ Live Gene—CTLGH, International Livestock Research Institute (ILRI), Addis Ababa, Ethiopia; ^4^ Centre for Tropical Livestock Genetics and Health (CTLGH), The Roslin Institute, The University of Edinburgh, Edinburgh, United Kingdom; ^5^ CAAS-ILRI Joint Laboratory on Livestock and Forage Genetic Resources, Institute of Animal Science, Chinese Academy of Agricultural Sciences (CAAS), Beijing, China; ^6^ Cells, Organism and Molecular Genetics, School of Life Sciences, University of Nottingham, Nottingham, United Kingdom

**Keywords:** Abergelle, Arado, Begait, Erob, private missense variants, Raya, runs of homozygosity, Tigray cattle

## Abstract

The Tigray region, where we found around eight per cent of the indigenous cattle population of Ethiopia, is considered as the historic centre of the country, with the ancient pre-Aksumite and Aksumite civilisations in contact with the civilisations of the Fertile Crescent and the Indian subcontinent. Here, we used whole genome sequencing data to characterise the genomic diversity, relatedness, and admixture of five cattle populations (Abergelle, Arado, Begait, Erob, and Raya) indigenous to the Tigray region of Ethiopia. We detected 28 to 29 million SNPs and 2.7 to 2.9 million indels in each population, of which 7% of SNPs and 34% of indels were novel. Functional annotation of the variants showed around 0.01% SNPs and 0.22%–0.27% indels in coding regions. Enrichment analysis of genes overlapping missense private SNPs revealed 20 significant GO terms and KEGG pathways that were shared by or specific to breeds. They included important genes associated with morphology (*SCN4A*, *TAS1R2* and *KCNG4*), milk yield (*GABRG1*), meat quality (*MMRN2*, *VWC2*), feed efficiency (*PCDH8* and *SLC26A3*), immune response (*LAMC1*, *PCDH18*, *CELSR1*, *TLR6* and *ITGA5*), heat resistance (*NPFFR1* and *HTR7*) and genes belonging to the olfactory gene family, which may be related to adaptation to harsh environments. Tigray indigenous cattle are very diverse. Their genome-wide average nucleotide diversity ranged from 0.0035 to 0.0036. The number of heterozygous SNPs was about 0.6–0.7 times higher than homozygous ones. The within-breed average number of ROHs ranged from 777.82 to 1000.45, with the average sum of the length of ROHs ranging from 122.01 Mbp to 163.88 Mbp. The genomic inbreeding coefficients differed among animals and breeds, reaching up to 10% in some Begait and Raya animals. Tigray indigenous cattle shared a common ancestry with Asian indicine (85.6%–88.7%) and African taurine (11.3%–14.1%) cattle, with very small, if any, European taurine introgression. This study identified high within-breed genetic diversity representing an opportunity for breeding improvement programs and, also, significant novel variants that could increase the number of known cattle variants, an important contribution to the knowledge of domestic cattle genetic diversity.

## Introduction

From the main domestication centres, cattle dispersed worldwide through trading and human migration routes (Hanotte et al., 2002; Beja-Pereira et al., 2006; Freeman et al., 2006; Ajmone-Marsan et al., 2010). The long process of their origin, domestication, and natural and artificial selection led to diversified phenotypic attributes related to their history, agro-ecologies and production systems (Ajmone-Marsan et al., 2010; Purfield et al., 2012; FAO, 2015). About 1019 local cattle breeds have been recognised worldwide (FAO, 2015). However, the growing demand for animal-based food products is resulting in the extensive introduction of a few specialised high-yielding milk and beef breeds (Ajmone-Marsan et al., 2010; FAO, 2015; Kukučková et al., 2017) with crossbreeding or replacement of the indigenous genotypes. It may trigger a sharp decline in the population size of local breeds (Medugorac et al., 2009) and erosion of their genetic makeup. It has been estimated that around 50% of the global cattle breeds’ diversity remains unknown (FAO, 2015). Characterising the diversity of indigenous breeds is important for understanding their adaptive traits and for targeted conservation strategies (FAO, 2015; Mwai et al., 2015; Addo et al., 2019; Eusebi et al., 2020).

Ethiopia is a major entry gate for cattle into the African continent (Hanotte et al., 2002; Li et al., 2007; Edea et al., 2015). It is the home of Africa’s largest cattle population and ranks the fifth worldwide (Mwai et al., 2015; CSA, 2018). It has 60.39 million heads of cattle, of which 98.24% are indigenous to the country (CSA, 2018) and managed by smallholder farmers (Rowlands et al., 2006; EBI, 2016). The indigenous cattle of Ethiopia produce, reproduce, and survive with little veterinarian intervention and limited feed resources, including in extreme temperatures (hot or cold) and diverse agro-ecologies ranging from low altitude (<500 m above sea level (m.a.s.l.) to high altitude mountainous areas (>3000 m.a.s.l.) (EBI, 2016; Bekuma and Hirpha, 2018).

The region of Tigray in the North of Ethiopia is an ancient centre of civilisations (e.g., ancient pre-Aksumite Kingdom of Da’amat and Aksumite Kingdom of Axum) which were in trading contacts with the ancient civilisations of the Fertile Crescent and the Indus Valley (Finneran, 2007; Pagani et al., 2012). Accordingly, it had an important role in the introduction of livestock into the Horn of Africa (Woldekiros and D’Andrea, 2017). 

Tigray is the fourth most cattle-populated Ethiopian region, with about 8% of the country’s cattle genetic resource (CSA, 2018). Previous studies have characterised some of these populations using low-density molecular markers such as microsatellites, *Y*-chromosome markers or SNPs arrays (Li et al., 2007; Zerabruk et al., 2007; Dadi et al., 2008; Zerabruk et al., 2011; Edea et al., 2015). Using five Y chromosome markers, Li et al. (2007) identified indicine but no taurine Y chromosome in the Tigray cattle with the exception of an Arado bull. Dadi et al. (2008) characterised the genetic diversity of Raya (Tigray cattle) and other cattle from different parts of Ethiopia using 30 microsatellite loci. Zerabruk et al. (2007) reported the genetic diversity of the five recognised Tigray cattle populations (Abergelle, Arado, Begait, Erob and Raya) using 20 autosomal microsatellite markers and observed that the Begait cattle had the highest within-population diversity among the examined ones. Using the same set of 20 autosomal microsatellite markers, Zerabruk et al. (2011) characterised the admixture composition of the Tigray cattle and reported a small proportion of European taurine background in some animals. Edea et al. (2015) genotyped three Tigray cattle populations (Arado, Begait and Raya) and four other Ethiopian cattle populations using the GeneSeek Genomic Profiler HD Bead Chip SNP array and found high genetic differentiation and unique admixture patterns in the Begait cattle.

Whole-genome sequence analyses are now the method of choice for genome diversity characterisation (Shendure and Ji, 2008; Stafuzza et al., 2017). At the opposite of microsatellite and SNPs arrays, they provide a complete representation of the diversity of a genome and an entry point to the identification of candidate causative variants associated with Mendelian and quantitative traits (Jiang et al., 2014; Das et al., 2015). Compared to SNPs arrays, often selected for polymorphisms in a reduced number of breeds, they are less prone to ascertainment biases. However, it should be noted that polymorphism detection relies on sequence alignment against a single genome of reference, which will still introduce biases in the identification of SNPs following the genome of reference chosen. Recently, a few studies (Kim et al., 2020; Jang et al., 2022; Terefe et al., 2022; Terefe et al., 2023) have reported the whole-genome characterisation of Ethiopian indigenous cattle, making it the African country with the largest number of cattle genome sequences available. However, they are still several main gaps in our knowledge with cattle populations from some geographic areas and cattle populations living in extreme environments yet to be characterised at the whole genome level (Edea et al., 2013; EBI, 2016). For instance, all the previous whole-genome based characterization studies on Ethiopian cattle populations (Kim et al., 2020; Jang et al., 2022; Terefe et al., 2022) did not include any Tigray cattle, with the exception of one Tigray cattle population (Begait) (Terefe et al., 2023).

We reported previously a multivariate morphological description of the Tigray cattle populations, using 21 qualitative traits and 21 body measurements (Zegeye et al., 2021). For the five Tigray indigenous cattle populations (Arado, Begait, Abergelle, Erob, and Raya cattle), four distinct clusters were identified with the Abergelle and Erob grouped together (Zegeye et al., 2021). Here, we characterised the same five populations using autosomal SNPs and insertion/deletion (indels) variants to assess their genetic diversity, differentiation, relatedness and admixture. We aimed to examine their genetic uniqueness and to pave the way for further analysis to identify genomic regions and, ultimately, the genetic control of their morphological and adaptative traits.

## Materials and methods

### Sample collection

**FIGURE 1:**
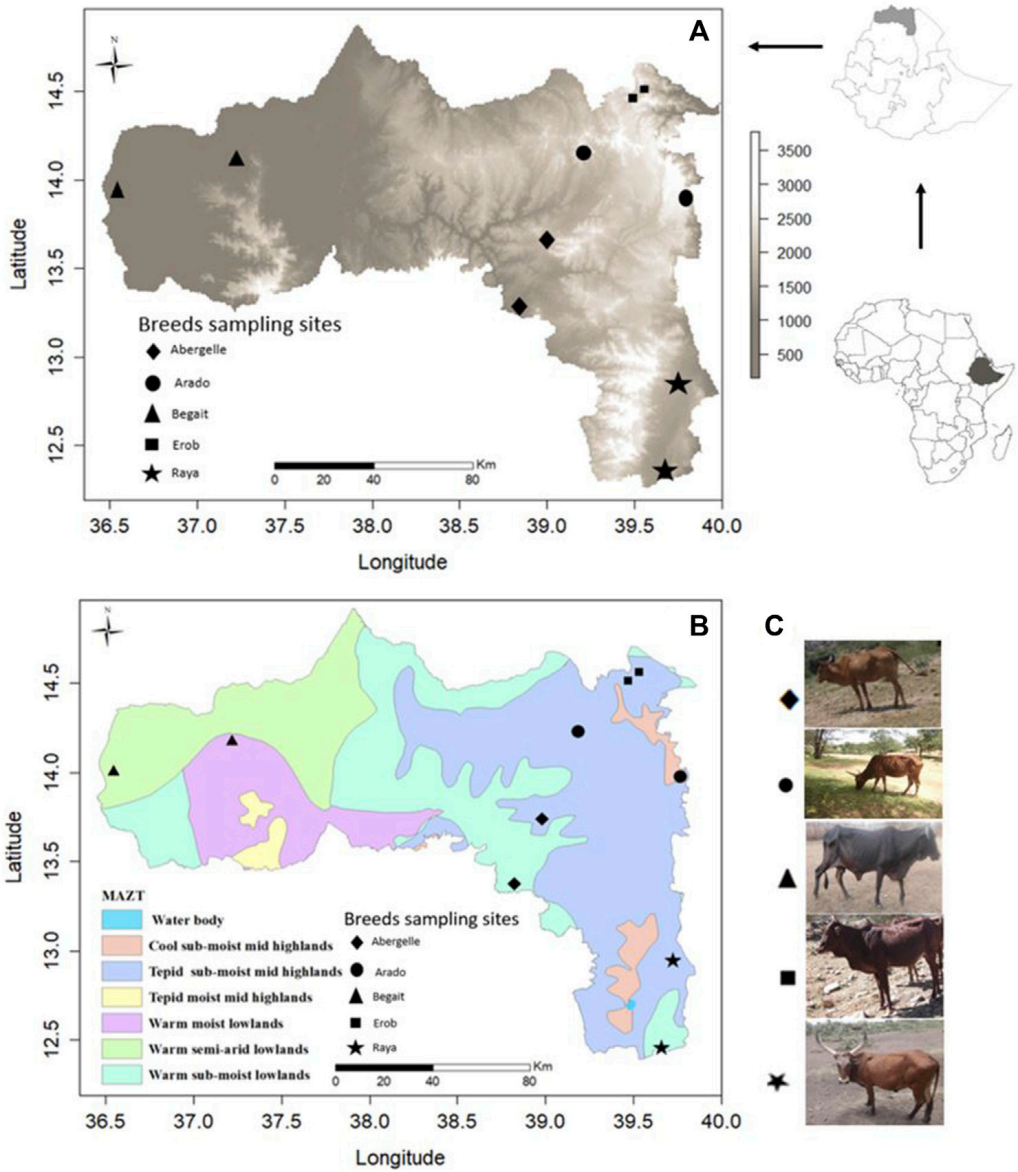
**(A)** Physical map of Tigray region based on elevation (meters above sea level, m.a.s.l.) with sampling sites for each population. **(B)** Physical map of Tigray region based on Major Agroecological Zones of Tigray (MAZT) (MOA, 1998) with sampling sites for each population. **(C)** Photos of the studied indigenous Tigray cattle populations.

Fifty-four whole blood samples were collected from five indigenous cattle populations (11 Abergelle, 11 Arado, 11 Begait, 10 Erob, and 11 Raya cattle) in the Tigray region of Northern Ethiopia (Figures 1A–C). The sampling area and morphological descriptions of the populations were reported previously (Zegeye et al., 2021). The whole blood was collected from the jugular vein of each animal by venipuncture with a 10 mL (millilitre) vacutainer blood collection tube containing ethylenediaminetetraacetic acid (EDTA) as an anticoagulant. The blood was gently mixed with the EDTA and placed into an icebox containing ice. It was brought to the International Livestock Research Institute molecular laboratory facility (ILRI - Addis Ababa), where it was stored at −21°C (degree centigrade) until the extraction of the genomic DNA (gDNA).

### Genomic DNA extraction and quality checking

The gDNA was extracted using the Qiagen DNeasy Blood and Tissue Kit (Qiagen, Hilden, Germany) following the manufacturer’s standard procedure. The extracted gDNA samples were evaluated for their concentration and quality using a Nanodrop spectrophotometer (DeNovix-DS-11+spectrophotometer, USA) and 1% agarose gel electrophoresis. A minimum of 5 µg of high quality gDNA with a concentration >50 nanogram per microliter (ng/µL) (Supplementary Table S1) was used for whole-genome sequencing.

### Library construction and sequencing

The gDNA samples were sent to the ILRI-CAAS Joint Laboratory of Livestock and Forage Genetic Resources in Beijing, P.R. China, which supervised the genome sequencing. Following the manufacturer’s specifications, a paired-end DNA library was constructed for each of the 54 samples. The gDNA was sequenced on an Illumina HiSeq X10 platform.

### Short read mapping and variant calling

The sequence reads were checked for quality using FastQC version 0.11.5. Following quality checking, paired-end reads (FASTAQ format) were aligned against the cattle reference genome assembly (ARS_UCD1.2, *Bos taurus*, Hereford breed) using the BWA version 0.7.17 (Li and Durbin, 2009). The mapped reads were sorted using samtools version 1.8 (Li et al., 2009) and converted to BAM formats using PICARD tools version 2.18.2. Duplicated reads were marked and removed using PICARD’s MarkDuplicates command. Moreover, the percentages of reads mapped to the reference genome were computed from dedup_recal.bam file using the Genome Analysis Toolkit’s (GATK, version 3.8-1-0-gf15c1c3ef) DepthOfCoverage “-ct 5 -ct 10 -ct 20 -ct 40”.

The base quality score recalibration (BQSR) was performed using the GATK’s BaseRecalibrator and the uniquely mapped reads for variant calling were selected using the GATK’s HaplotypeCaller (McKenna et al., 2010). The genomic variants (GVCF files) generated from each sample were jointly analysed using the GATK’s GenotypeGVCFtool. Called variants (SNPs and indels) were separately subjected to variant filtration (GATK hard filter) setting MQ > 40, QD > 2.0, ReadPosRankSum > 8.0, MappingQualityRankSum > 12.5 and HaplotypeScore > 13 for SNPs and FS > 200.0, QD < 2.0, ReadPosRankSum < −20.0 and QUAL <20) for indels. Only bi-allelic variants that meet the specified filtering criteria were selected for further analysis.

### Variant statistics and annotation

To compute the variant statistics (e.g., total number of SNPs, total number of indels, indel length, and nucleotide substitution), we used the VCF-stats command of VCFtools/0.1.14/Perl. The number of transition and transversion, average ratios of transitions-to-transversions (Ti/Tv), and distribution of SNPs and indels at different allelic frequencies were analysed using stats command and plot-VCF-stats of BCFtools/1.8 (Li et al., 2009). Moreover, SNPs and indels density across chromosomes were computed for each population using VCFtools version 0.1.15 and then averaged using R version 3.6.1 (R Development Core Team, 2019). We searched and compared our SNPs against the dbSNP *ver*150 (https://genome.ucsc.edu/cgi-bin/hgGateway, last accessed in July 2021). Finally, the variants (SNPs and indels) were classified according to their potential functions using the Ensemble Variant Effect Predictor tool (VEP, (https://www.ensembl.org/info/docs/tools/vep/index.html), and the genes overlapping private missense variants were functionally annotated by DAVID version 6.8 (https://david.ncifcrf.gov/home.jsp). Significant Gene Ontology (GO) and Kyoto Encyclopedia of Genes and Genomes (KEGG) pathways were selected based on different criteria including *p* < 0.05, Bonferroni < 0.05, FDR < 0.05 and fold enrichment > 1.

### Genome-wide nucleotide diversity and heterozygosity

The genome-wide nucleotide diversity (*π*) was analysed for each population using VCFtools version 0.1.15 in 20 kb windows with a 10 kb sliding step (with the--window-pi 20000 --window-pi-step 10000 option) (Danecek et al., 2011). The numbers of non-reference heterozygous and homozygous variants (SNPs and indels) were analysed using the VCF-stats command of VCFtools/0.1.14/Perl. Further, the observed heterozygosity (*Ho*) was calculated following the command “--het” in PLINK version 1.9 (Purcell et al., 2007).

### Runs of homozygosity and genomic inbreeding

The runs of homozygosity (ROH) were detected using PLINK version 1.9 (Purcell et al., 2007) by setting a sliding window of 50 SNPs (--homozyg-window-snp 50), one possible heterozygous genotype (--homozyg-window-het 1), two missing genotypes (--homozyg-window-missing 2), a minimum SNP density of 1 SNP every 50 kb (--homozyg-density 50), a minimum number of 100 SNPs (--homozyg-snp100), a minimum length of 100 kb (--homozyg-kb 100), a maximum gap of 1 Mb between consecutive homozygous SNPs (--homozyg-gap 1000) and the presence of the SNP in at least five homozygous reads (--homozyg-window-threshold 0.05).

We calculated the genomic inbreeding value for each cattle by dividing the sum of ROHs length with the total length of the genome (F_ROH_ = LROH/LAUTO), following McQuillan et al. (2008), Zhang et al. (2015a), Addo et al. (2019) and Guo J. et al. (2019). For this analysis, we considered a total genome length of 2,715,853,792 bp (2.72 Gb) (ARS_UCD1.2). An alternate inbreeding coefficient (F_HOM_) was also calculated for each animal using the “--het” command in PLINK version 1.9 (Purcell et al., 2007) following Addo et al. (2019).

### Genetic relationship and differentiation

Publicly available genome sequences of 15 cattle breeds (Supplementary Table S2) from six reference groups were added to the dataset for genetic relationship and differentiation analyses. These included African sanga (Afar and Ankole, crosses between African zebu and longhorn humpless taurine), African zenga (Fogera and Horro, crosses between African zebu and sanga), African zebu (Ethiopian Boran and Kenana), African taurine (Muturu and N’Dama), European taurine (Angus and Holstein), Asian zebu (Bhagnari, Cholistani, Dhanni, Sahiwal and Tharparkar). The VCFs of all the reference populations were generated from their raw sequence reads by applying the same procedures mentioned above, and subsequently merged with the five Tigray cattle populations. The merged dataset included 164 cattle genomes and 42,766,398 raw SNPs. It was pruned using PLINK version 1.9 (Purcell et al., 2007) by setting different filtering and quality control thresholds, such as “--mind 0.25 --geno 0.1 --maf 0.05 --indep-pairwise 50 10 0.5 --set-missing-var-ids C@P”. Where--mind 0.25 = individual sample to be removed following 25% or more missing genotype data, --geno 0.1 = variants to be removed due to 10% of missing genotype data, --maf 0.05 = variants to be removed due to minor allele frequency less than 0.05, --indep-pairwise 50 10 0.5 = SNPs with pairwise *r* ^2^ values higher than 0.5 in sliding windows of 50 SNPs moving stepwise with ten SNPs at a time across the genome and set-missing-var-ids C@P = missing IDs set. After applying the quality control and filtering thresholds, the pruned final data set including 3,695,054 SNPs and 164 animals was converted to plink. fam, plink. bin, and plink. bed file using the flag “--make-bed” in PLINK version 1.9 (Purcell et al., 2007).

#### Principal component analysis

The LD-pruned dataset consisting of 3,695,054 SNPs and 164 individuals was used for principal component analysis (PCA). To calculate pca.egenvel and pca.egenvec, we used the flag “plink--pca” with a default parameter, for the first 20 principal components (PCs). Then, the proportions of variances explained by the eigenvector were computed by dividing each egenvel by the total sum of all egenvels (1–20) and expressing it as a per centage. Finally, the two first PCs were plotted against each other using the ggplot2 package in R version 3.6.1 (R Development Core Team, 2019) to illustrate the population clustering.

#### Genetic admixture analysis

Using the same LD-pruned dataset (3,695,054 SNPs), the ADMIXTURE version 1.3.0 software (Alexander et al., 2009) was used to determine the optimal number of clusters (K) and to describe individual ancestry. A cross-validation procedure was performed using the program’s flag "-cv” for K = 1 to K = 10. The K with the lowest cross-validation error was taken as the recommended number of clusters for the dataset. The cross-validation error value for each K (1–10) and the cluster assignments were plotted using R version 3.6.1 (R Development Core Team, 2019).

#### Genetic differentiation

The genetic distance (*F*_ST_) between pairs of populations (Weir and Clark Cockerham, 1984) was analysed using VCFtools version 0.1.15 in 100 kb windows with a 50 kb sliding step (with the--window-pi 100000 --window-pi-step 50000 option) (Purcell et al., 2007). The pairwise weighted *F*_ST_-*based* heat map with a dendrogram was plotted in R version 3.6.1 (R Development Core Team, 2019). Next, a Neighbor-Net tree based on pairwise *F*_ST_ values was constructed using the Neighbor-Net algorithm (Bryant and Moulton, 2004) implemented in SplitsTree5 V 5.0.0” (Huson and Bryant, 2006) and plotted in R version 3.6.1 (R Development Core Team, 2019).

## Results

### Intra-population genetic diversity in the Tigray cattle

#### Mapping and variant detection

The number of paired-sequence reads for each animal ranged from 200,684,387 to 289,752,799, with a mapping rate of 99.61%–99.79% to the reference genome (ARS_UCD1.2). The average sequencing depth among populations ranged from 10.13 X (Erob cattle) to 10.64 X (Begait cattle). Furthermore, over 88% of the bases were covered with at least five reads, and 39%–42% were covered with at least ten reads (Supplementary Table S3).

Variant calling and filtration combining the five Tigray cattle populations resulted in the detection of around 36 million (M) SNPs (n = 36,003,573) and 3.7 M indels (n = 3,703,659) (Supplementary Table S4B). The number of SNPs detected per individual sample ranged from 12 M to 13 M (Supplementary Table S4A). The number of SNPs at population level ranged from 28 M to 29 M, of which 7% were novel (Table 1). A total of 2,113,093 (7.15%) SNPs were shared among Abergelle cattle, 2,062,642 (6.94%) among Arado cattle, 2,182,704 (7.54%) among Begait cattle, 2724,442 (9.71%) among Erob cattle, and 2,161,735 (7.35%) among Raya cattle (Supplementary Table S4B). Around 674,019 (1.87%) SNPs were shared across the five Tigray cattle populations (Supplementary Table S4B).

**TABLE 1:** Variant statistics within cattle populations from Tigray region, Ethiopia.

Variables	Cattle breeds				
	Abergelle	Arado	Begait	Erob	Raya
No. of samples	11	11	11	10	11
SNPs					
Novel (%)	2135111 (7.22)	2138760 (7.2)	2091767 (7.23)	1999834 (7.13)	2161976 (7.36)
Known	27428853	27574364	26840780	26045211	27231606
Total	29563964	29713124	28932547	28045045	29393582
Indels					
Novel (%)	985169 (34.11)	991807 (34.13)	968237 (34.22)	938479 (34.06)	1003423 (34.6)
Known	1902736	1914050	1861562	1817017	1896421
Total	2887905	2905857	2829799	2755496	2899844

**No. = number, this abbreviation works for all No. in the tables.**

We detected around 1.1 M–1.2 M indels in each individual cattle (Supplementary Table S4A), while the number of indels in each population ranged from 2,755,496 (Erob) to 2,905,857 (Arado). Of these, around 34% were novel (Table 1). Within a population, 177,353 (6.14%), 173,842 (5.98%), 183,302 (6.48%), 228,704 (8.3%) and 181,610 (6.26%) indels were common to all samples in Abergelle, Arado, Begait, Erob, and Raya cattle populations, respectively. Around 1.43% (52,992) of indels were shared across all the five Tigray cattle populations (Supplementary Table S4B).

**FIGURE 2:**
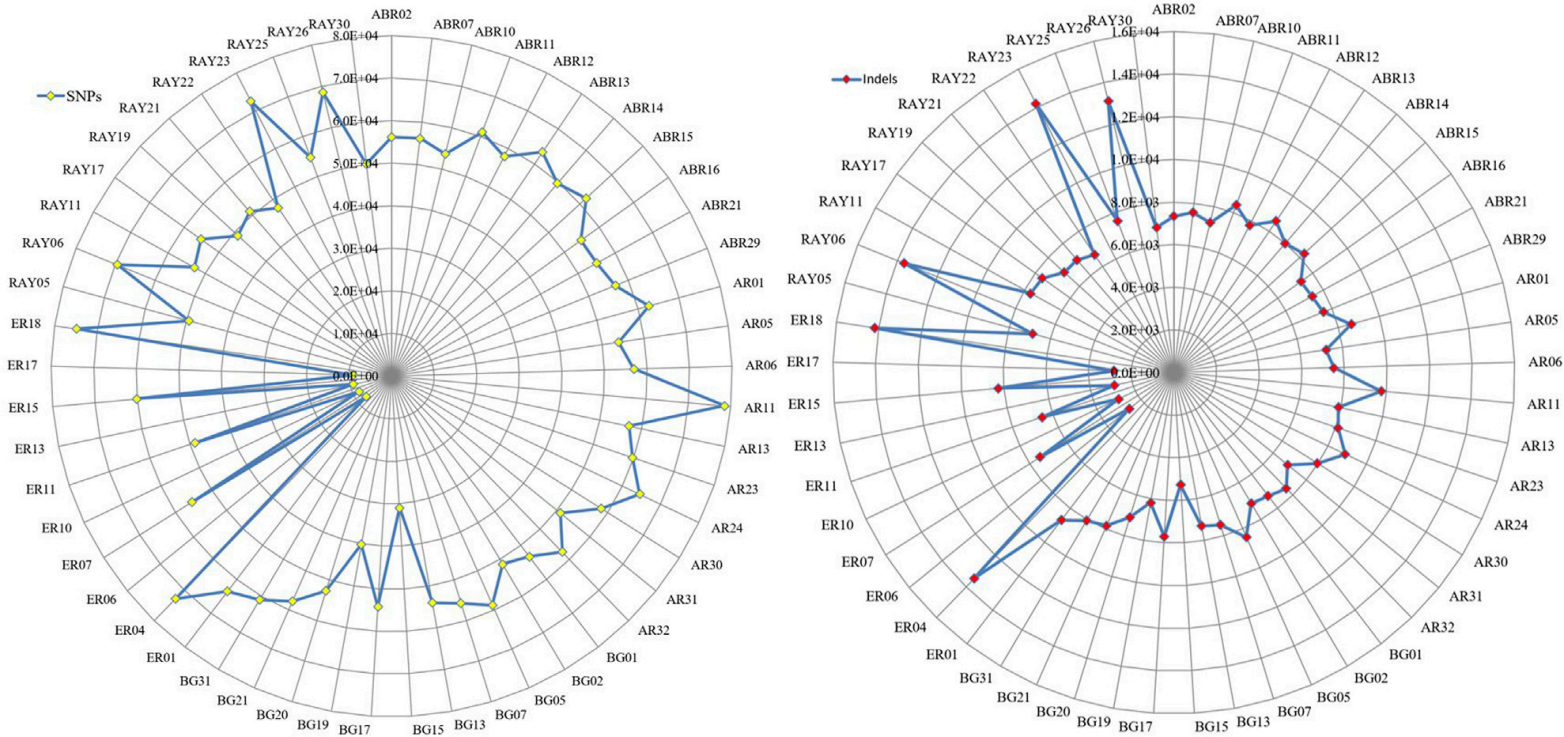
The distribution of private SNPs and indels across samples for each population (ABR* for Abergelle, AR* for Arado, BG* for Begait, ER* for Erob and RAY* for Raya cattle).

Except for Erob cattle, the number of private SNPs across individual samples ranged from 32,245 to 81,933, and the number of private indels ranged from 5,276 to 14,182 (Figure 2). Among Erob cattle, four samples (ER06, ER17, ER13 and ER10) had fewer private variants (9,929 to 11,526 SNPs and 2,675 to 2,870 indels) compared to the remaining Tigray cattle samples. At the population level, we detected 571,535, 634,275, 583,831, 433,013 and 569,013 private SNPs for Abergelle, Arado, Begait, Erob and Raya cattle populations, respectively.

##### Density of the variants and their allele frequencies

The density of genome-wide SNPs ranged from 11.27 ± 7.69 to 11.94 ± 7.88 SNPs/kb and of indels from 1.08 ± 1.34 to 1.17 ± 1.41 indels/kb across the five Tigray cattle populations (Supplementary Tables S5, S6). Chromosomes 23, 27 and 28 had the highest density of variants (13–14 SNPs/kb and 1.3 to 1.4 indel/kb), while chromosomes 19, 13, 3 and 11 had the lowest ones (10–11 SNPs/kb and <1.1 indels/kb). The chromosome-wise distributions of variants (SNPs and indels) were proportional to the length of the chromosomes (Supplementary Tables S7, S8). As expected, large chromosomes had more variants than small ones (Supplementary Figures S1A–D). However, the density of variants (SNPs/kb or indel/kb) was higher on small chromosomes than large ones.

**FIGURE 3:**
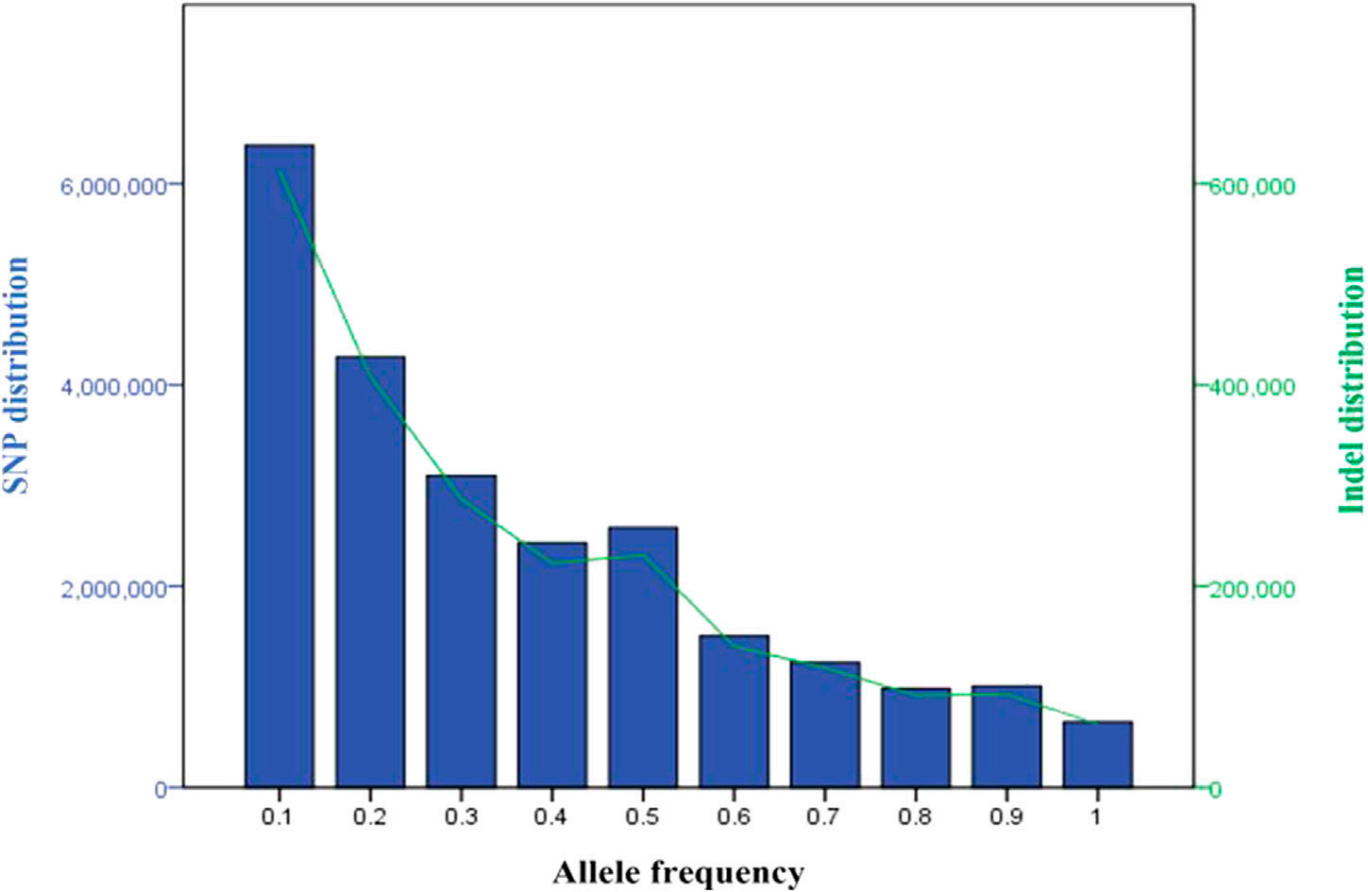
Distribution of the variants based on allele frequency (blue bars represent SNPs and the green line represents indels).

**FIGURE 4:**
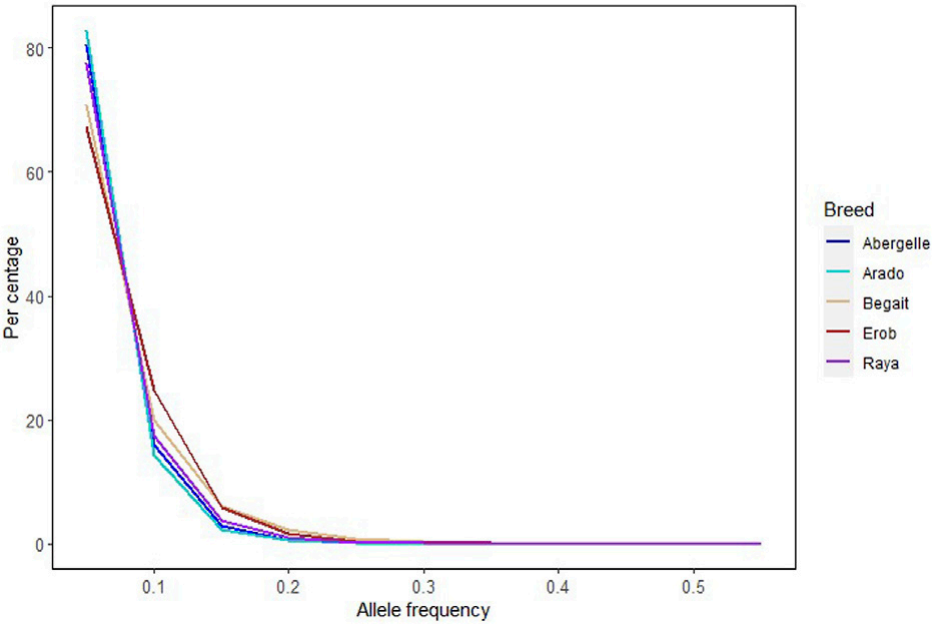
Allele frequency of private SNPs, where each coloured line represents a cattle population.

Across the five Tigray indigenous cattle populations, the average alternate (non-reference) allele frequencies of SNPs and indels were 0.32 and 0.28 to 0.3, respectively. The proportion of SNPs with mean alternate allele frequency (AAF) < 0.5 ranged from 77% to 79% and the proportion of SNPs with mean AAF > 0.9 was around 4%. The proportion of indels with mean AAF < 0.5 ranged from 78% to 80% (Supplementary Table S9). However, most of the variants (SNPs and indels) had frequencies of 10% or less (Figure 3). Allele frequencies of private SNPs ranged from 0.05 to 0.55, of which 67% (Erob cattle) to 83% (Arado cattle) of these SNPs had an allele frequency of 0.05 (Figure 4).

##### Nucleotide substitutions and indel length

The Ti/Tv ratio was around 2.35 (Supplementary Table S10). It supported a high sequencing accuracy for all samples. Across all the samples, the highest number of nucleotide substitutions were recorded for the bases Cytosine to Thymine (C > T) and the bases Guanine to Adenine (G > A) while the least number of nucleotide substitutions for the bases Adenine to Thymine (A > T) and Thymine to Adenine (T > A) (Supplementary Table S10 and Supplementary Figure S2A). The number of insertions was about 0.33–0.35 times higher than deletions. Furthermore, the length of indels ranged from −28 bp (deletion, Abergelle) to +23 bp (insertion, Begait) (Supplementary Table S11). Almost 50% of the indels had a length of 1 bp, while the majority of indels were less than 5 bp (87.12% in Abergelle, 87.08% in Arado, 87.11% in Begait, 87.18% in Erob and 87.11% in Raya cattle). Only around 13% of the total indels had lengths greater than or equal to 6 bp (Supplementary Table S11 and Supplementary Figure S2B).

#### Functional distribution of variants (SNPs and indels)

The annotation of the SNPs showed that around 59.5% of them were in the intergenic regions. Around 76% of annotated SNPs were in introns, 7% in upstream of genes, 7.2% in downstream of genes, 0.5% in 3′ untranslated region (UTR), 0.2% in 5’ UTR and 0.11% in non-coding transcript exon. The number of SNPs in the coding regions (stop gain, stop lost and stop retained, start lost, missense and synonymous SNPs, and coding sequences) was approximately 0.01% in all populations (380,309 in Abergelle, 383,116 in Arado, 373,578 in Begait, 361,55 in Erob and 380,299 in Raya cattle) (Table 2) (Supplementary Figure S3), of which around 17%–18% had deleterious effects (Supplementary Figure S4).

**TABLE 2:** Population level summary of annotation of SNPs in the Tigray cattle.

All consequences	Abergelle	Arado	Begait	Erob	Raya
No. of samples	11	11	11	10	11
Bi-allelic variants processed	29563964	29713124	28932547	28045045	29393582
Splice donor variant	707	684	693	672	690
Splice acceptor variant	439	452	442	429	435
Stop gained	1682	1748	1706	1648	1765
Stop lost	245	238	237	239	243
Start lost	397	406	382	373	413
Missense variant	149334	150764	146883	141733	149448
Splice region variant	40755	41135	39987	38861	40791
Synonymous variant	228481	229776	224200	217399	228249
Stop retained variant	169	183	169	160	180
Coding sequence variant	1	1	1	1	1
Mature miRNA variant	123	125	121	120	125
5_prime UTR variant	49794	49766	48751	46913	50016
3_prime UTR variant	137652	138671	135235	131341	137754
Non-coding transcript exon variant	31400	31309	30515	29622	31084
Intron variant	22387867	22473356	21940886	21255569	22194958
Non-coding transcript variant	502772	505660	496259	476848	500512
Upstream gene variant	2074721	2090124	2042625	1979084	2075740
Downstream gene variant	2111713	2121229	2070242	2007161	2104907
Intergenic variant	17584768	17682416	17197428	16677542	17502131

Around 57%, 77%, 7.4%, 8%, 0.6%, 0.2% and 0.1% of the indels were in intergenic regions, introns, upstream of genes, downstream of genes, 3′ UTR, 5’ UTR and non-coding transcript exons, respectively. The total numbers of indels located within the coding regions (stop gain, stop lost, stop retained, start lost, start retained, frameshifts, inframe insertions, inframe deletions, protein-altering variants and coding sequences) ranged from 0.22% (Raya cattle, 6,414) to 0.27% (Erob cattle, 7,462) (Table 3). Among the indels located in the coding regions, 63.20%, 63.39%, 62.65%, 63.48% and 77.6% resulted in codon frameshifts (codon alteration), of which 0.81%, 0.84%, 0.98%, 0.84% and 0.88% may affect protein functions in Abergelle, Arado, Begait, Erob, and Raya cattle populations, respectively (Supplementary Figure S5).

**TABLE 3:** Population level summary of annotation of indels in the Tigray cattle.

All consequences	Abergelle	Arado	Begait	Erob	Raya
No. of samples	11	11	11	10	11
Bi-allelic variants processed	2887905	2905857	2829799	2755496	2899844
Transcript ablation	4	5	5	5	5
Splice donor variant	248	240	236	206	227
Splice acceptor variant	232	234	215	208	221
Stop gained	57	61	64	62	61
Frameshift variant	4788	4875	4688	4737	4977
Stop lost	28	28	28	24	25
Start lost	42	39	41	34	38
Inframe insertion	825	834	815	799	853
Inframe deletion	1565	1578	1561	1540	1781
Protein altering variant	39	41	46	40	44
Splice region variant	3797	3913	3805	3716	3952
Stop retained variant	17	17	21	22	20
Start retained variant	13	14	13	13	12
Coding sequence variant	202	204	206	191	206
Mature miRNA variant	12	16	13	16	17
5 prime UTR variant	4855	4835	4842	4647	4985
3 prime UTR variant	16000	16025	15703	15327	16165
Non-coding transcript exon variant	2439	2440	2409	2321	2441
Intron variant	2237586	2246670	2192419	2135981	2239005
Non-coding transcript variant	47834	48290	47388	45827	48017
Upstream gene variant	214934	216603	211424	205980	217097
Downstream gene variant	230871	232624	227186	220614	232751
Intergenic variant	1642232	1653813	1607488	1565117	1648903

##### Enrichment analysis of the genes overlapping private SNPs

**FIGURE 5:**
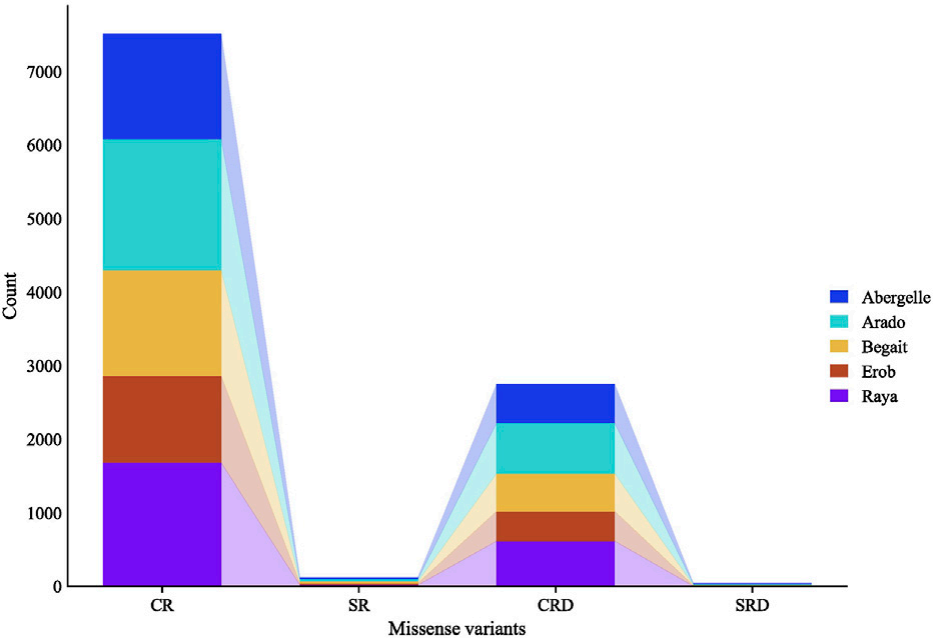
Private SNPs with missense effects overlapping coding and splicing regions, where, CR represents the number of missense SNPs overlapping coding regions, SR represents the number of missense SNPs overlapping splice regions, CRD represents the number of missense SNPs overlapping coding regions with deleterious effects, and SRD represents the number of missense SNPs overlapping splice regions with deleterious effects.

A separate analysis of the private SNPs for each population showed 1,455, 1,809, 1,470, 1,203, and 1,701 private missense SNPs for Abergelle, Arado, Begait, Erob and Raya cattle populations, respectively; of which 97.9% (Erob) to 98.9% (Raya) were in coding regions while the remaining ones overlapped with splice regions. Of the missense SNPs in the coding regions, 33.1%, 34.4%, 52.7%, 64.8% and 58.1% had deleterious effects in Abergelle, Arado, Begait, Erob and Raya cattle populations, respectively (Figure 5).

**FIGURE 6:**
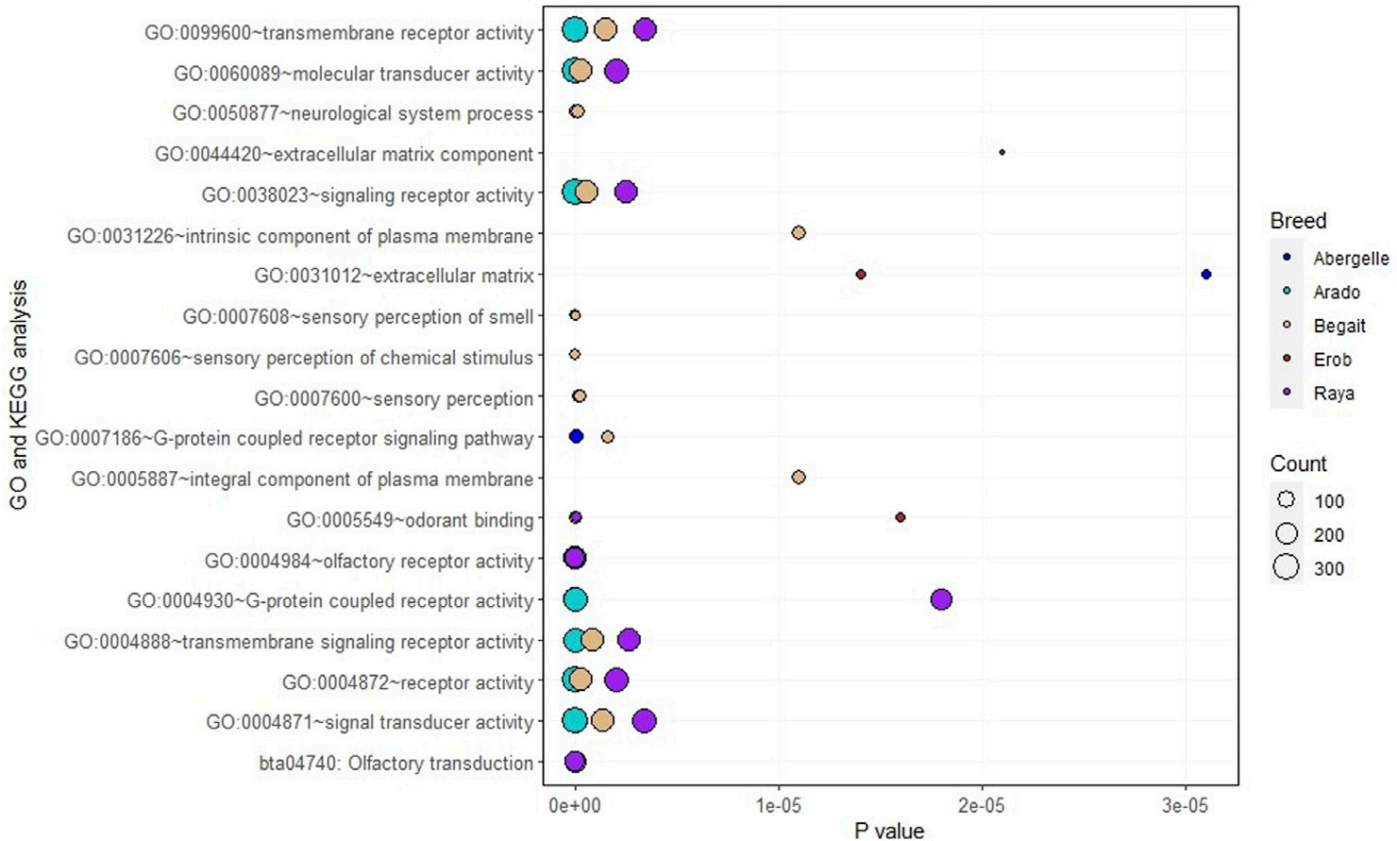
Gene Ontology (GO) and Kyoto Encyclopedia of Genes and Genomes (KEGG) pathway terms in the Tigray cattle, where the size of the circles represents how large the number of genes represented in a specific GO or KEGG pathway term and the level of significance, while each coloured circle represents a cattle population.

Functional enrichment analysis of the genes overlapping private SNPs in coding regions identified 16, 12, 16, 6 and 10 significant (*p* < 0.05, Bonferroni < 0.05, FDR < 0.05 and fold enrichment > 1) GO terms of biological process (BP), cellular component (CC), molecular function (MF) and KEGG pathways in Abergelle, Arado, Begait, Erob and Raya cattle populations, respectively (Figure 6). Out of the enriched terms, the top three most significant ones (*p* = 1.6 × 10^−5^ to 6.8 × 10^−67^, Bonferroni = 1.65 × 10^−2^ to 7.5 × 10^−64^, FDR = 8.3 × 10^−3^ to 7.5 × 10^−64^ and fold enrichment = 2.3–4.3) were olfactory receptor activity (GO:0004984), olfactory transduction (bta04740) and odorant binding (GO:0005549). These were common to all five Tigray cattle populations.

Within populations, the enrichment analysis further identified many population specific genes associated with the aforementioned three most significant terms. Around 11% (Erob cattle) to 17% (Abergelle cattle) of the genes were associated with olfactory receptor activity, 13% (Erob cattle) to 18% (Abergelle cattle) with olfactory transduction, and 3% (Erob cattle) to 6% (Abergelle cattle) with odorant binding (Additional file, Sheet 1).

Eleven genes, including *OR4F73*, *OR1L21*, *OR5AN1*, *OR9S29*, *OR9M1D*, *OR2H20*, *OR4X16*, *OR5AK29*, *OR6C4*, *OR8B1AU* and *OR9S40*, were commonly enriched in the three shared GO terms. Six GO terms (GO:0004871∼signal transducer activity, GO:0004872∼receptor activity, GO:0004888∼transmembrane signaling receptor activity, GO:0038023∼signaling receptor activity, GO:0060089∼molecular transducer activity, GO:0099600∼transmembrane receptor activity) with related molecular functions were enriched in four populations (Abergelle, Arado, Begait and Raya cattle). Interestingly, two significant GO terms of the cellular component associated with an integral component of the plasma membrane (GO:0005887) and the intrinsic component of the plasma membrane (GO:0031226) were only enriched in Begait cattle. Two GO terms of the cellular component (GO:0005578) related with proteinaceous extracellular matrix and extracellular matrix component (GO:0044420) were significantly enriched only in the Erob cattle (Additional file, Sheet 1).

#### Nucleotide diversity and heterozygosity

**FIGURE 7:**
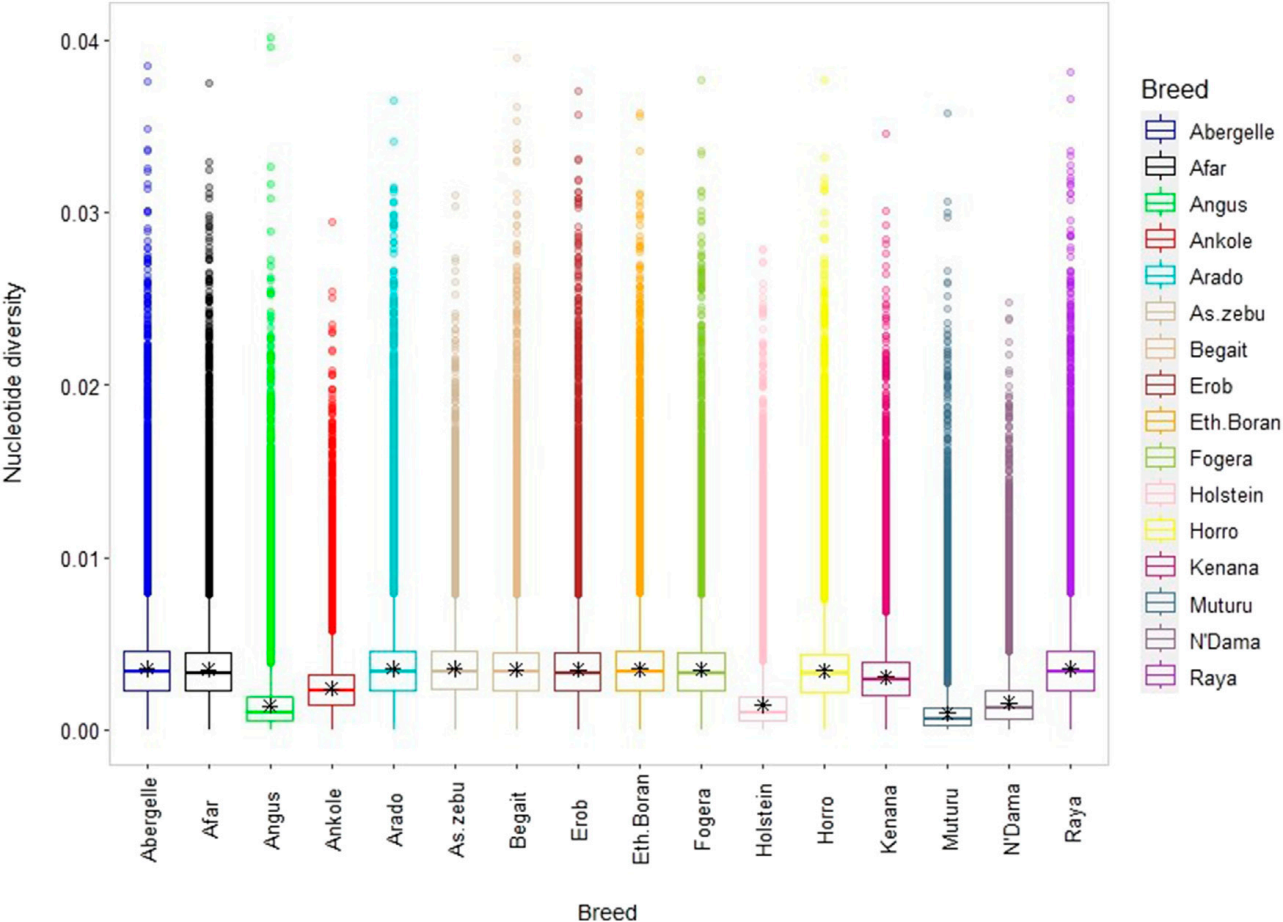
Box plot for nucleotide diversity, where each coloured box plot represents a cattle population.

The average genome-wide nucleotide diversity (*π*) ranged from 3.5 ± 1.77 × 10^−3^ (Raya) to 3.57 ± 1.76 × 10^−3^ (Arado) (Figure 7 and Supplementary Table S12). Besides, the average non-reference heterozygous variants (SNPs and indels) were around 0.6 to 0.7 times higher than the corresponding homozygous variants (Supplementary Table S12). At the individual level, the ratio of heterozygous to homozygous SNPs ranged from 1.39 to 1.89, and of the indels from 1.39 to 1.84 (Supplementary Figures S6A–E). The mean observed heterozygosity (*Ho*) was the highest in Arado cattle (0.302 ± 0.010) but the lowest in Raya cattle (0.278 ± 0.016) (Supplementary Table S12).

#### Runs of homozygosity and genomic inbreeding

##### Abundance and length of ROH in the Tigray cattle compared to major reference cattle groups

**FIGURE 8:**
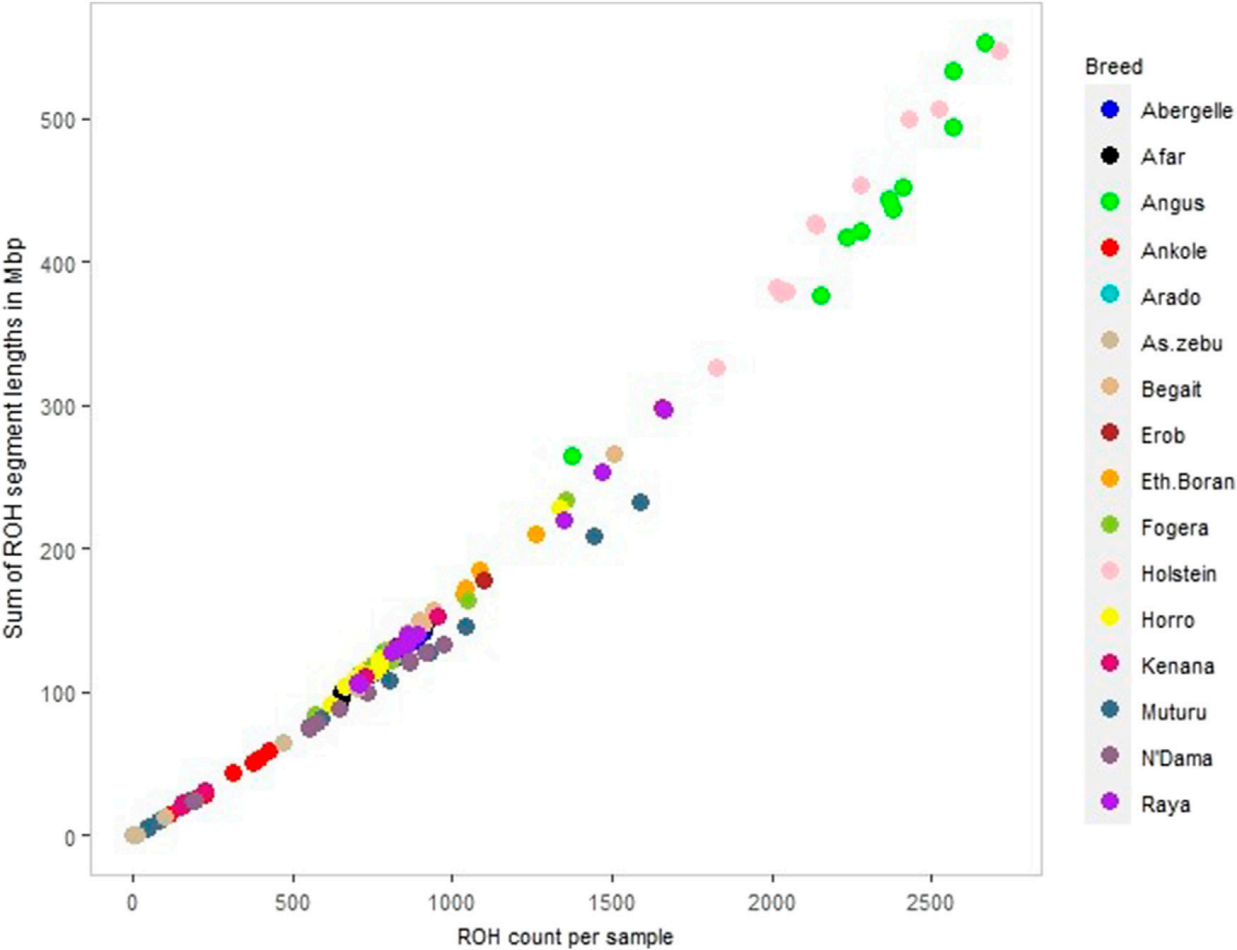
ROH profile of each animal across all cattle, including reference populations, where individual colour and circle represent a cattle population and individuals, respectively.

We calculated ROH for the five Tigray cattle populations and the reference breeds. The average number and length of ROH segments varied considerably within and among breeds (Figure 8; Table 4 and Supplementary Table S13). The within breed average number of ROH for the Tigray cattle ranged from 777.82 (Arado cattle) to 1000.45 (Raya cattle), and the within breed average sum of the length of ROH ranges from 122.01 megabase pairs (Mbp) (Arado cattle) to 163.88 Mbp (Raya cattle). The average number of ROHs and the average sum of the length of ROH in the Tigray cattle were higher than the ones recorded in Asian zebu, African zebu from Sudan (Kenana), African taurine (Muturu and N’Dama) and African sanga from Uganda (Ankole). But they were much lower compared to European taurine cattle (Holstein and Angus). However, in general, all the Tigray cattle had close ROH profiles with the other cattle populations originating from Ethiopia (Afar, Eth. Boran, Fogera and Horro) (Table 4).

**TABLE 4:** Number of animals with and without ROH, breed-wise total and average number of ROH and average sum of the length of ROH for the Tigray cattle populations compared to reference cattle populations.

Breed	No. w/o ROH^a^	No. w/ROH^b^	Total no. of ROH^c^	Avg. no. of ROH segments (min-max)^d^	Avg. ROH segment lengths in Mbp (min-max)^e^
Abergelle	0	11	9131	830.09 (748–907)	130.13 (112.92–148.83)
Arado	0	11	8556	777.82 (706–861)	122.01 (108.02–135.65)
Begait	0	11	10003	909.36 (767–1504)	148.9 (121.14–266.71)
Erob	0	10	8371	837.1 (727–1099)	131.92 (110.40–177.46)
Raya	0	11	11005	1000.45 (703–1662)	163.88 (105.87–297.29)
Eth.Boran	0	10	8937	893.7 (734–1259)	143.94 (113.6–210.41)
Kenana	0	10	4624	462.4 (150–1657)	73.79 (20.24–298.06)
Fogera	0	9	7817	868.56 (571–1353)	124.5 (84.47–234.48)
Horro	0	11	8508	773.45 (618–1334)	121.74 (91.91–228.27)
Ankole	0	10	2299	209 (6–423)	31.16 (0.78–58.99)
Afar	0	10	7441	744.1 (649–918)	115.21 (95.06–146.82)
Holstein	0	10	22124	2212.4 (1827–2710)	432.61 (325.89–547.33)
Angus	0	10	22999	2299.9 (1374–2670)	439.31 (264.38–553.70)
Muturu	0	10	6755	675.5 (40–1590)	94.88 (4.79–232.72)
N’Dama	0	10	6870	687 (194–969)	94.49 (23.67–133.10)
Asian zebu	1	9	1292	143.56 (0–704)	20.39 (113.57–103.09)

^a^ Number of animals without ROH.

^b^ Number of animals with ROH.

^c^ Total number of ROH across each population.

^d^ Average number of ROH segments (Minimum to maximum).

^e^ Average ROH segment length in megabyte (Minimum to maximum).

##### Distribution of ROH based on segment length categories

The number of ROH across length categories (0.1–0.25 Mbp, > 0.25–0.5 Mbp, > 0.5–1 Mbp and > 1 Mbp) varied among breeds. ROH in the length category of 0.1–0.25 Mbp accounted for 90%–92% of the total ROH. For the length categories >0.25–0.5 Mbp and > 0.5–1 Mbp, ROH frequencies were about 7%–10% and 0.2%–0.4%, respectively (Supplementary Tables S14, S15). ROH > 1 Mbp were only found in Begait and Raya cattle (BG15, RAY11, RAY22 and RAY26) (Supplementary Table S13). In the length categories 0.1–0.25 Mbp, the Tigray cattle had more ROH when compared to Holstein and Angus, but they were less than the African sanga from Uganda (Ankole), African taurine (Muturu and N’Dama) and the Asian zebu. Above the 0.25 Mbp length category, the Tigray cattle had more ROH when compared to Ankole, Muturu, N’Dama and Asian zebu, but they were less than Angus and Holstein (Supplementary Tables S14, S15).

##### Genomic positions under runs of homozygosity and inbreeding across the Tigray cattle

**FIGURE 9:**
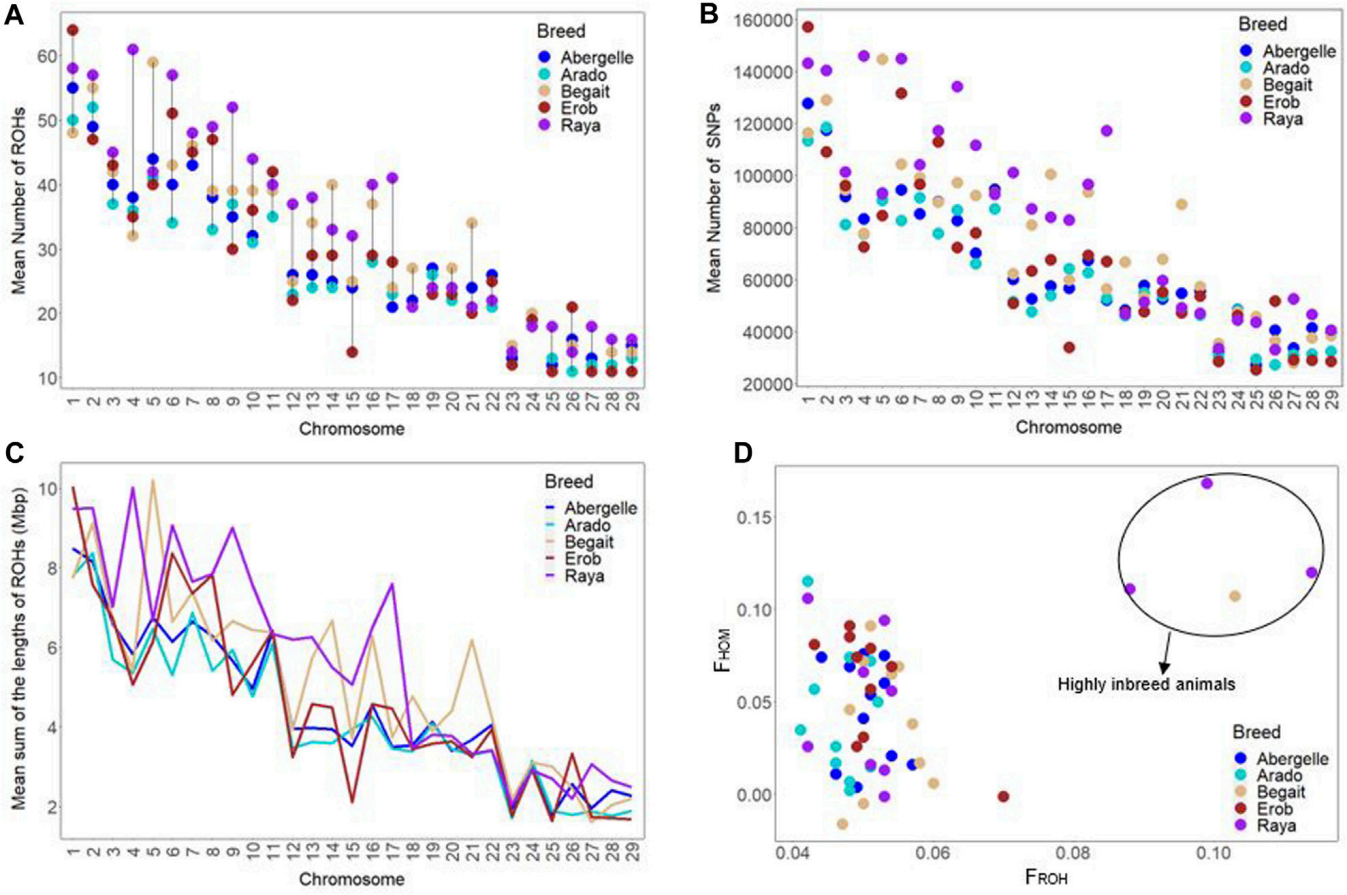
Genomic positions under runs of homozygosity (ROH). **(A)** Chromosome-wise mean number of ROH across cattle populations. **(B)** Chromosome-wise mean sum of ROH lengths across cattle populations. **(C)** Incidence of SNPs on ROH across each autosome among individual animals of each cattle population (where each circle represents an individual within a population and each colour represents a population). **(D)** Inbreeding coefficients (F_ROH_ and F_HOM)_ among individual animals of each cattle population (where each circle represents an individual within a population and each colour represents a population).

The chromosome-wise distributions of the number and length of ROH and the incidence of SNPs on ROH were different across the five Tigray cattle populations, except for the Abergelle and Erob cattle (Figures 9A–C and Supplementary Tables S16–S18). At the population level, the mean genomic inbreeding coefficient was the smallest in Arado (F_ROH_ = 0.047 ± 0.004 and F_HOM_ = 0.043 ± 0.035) but the highest in Raya cattle (F_ROH_ = 0.064 ± 0.025 and F_HOM_ = 0.07 ± 0.054) (Supplementary Table S19). One Begait cattle (BG15 with F_ROH_ = 0.103 and F_HOM_ = 0.107) and three Raya cattle (RAY11 with F_ROH_ = 0.114 and F_HOM_ = 0.12, RAY22 with F_ROH_ = 0.099 and F_HOM_ = 0.168, and RAY26 with F_ROH_ = 0.088 and F_HOM_ = 0.111) showed strong genomic inbreeding values based on both methods (Figure 9D and Supplementary Table S20).

### Relationship and inter-population genetic differentiation

#### Principal component analysis

**FIGURE 10:**
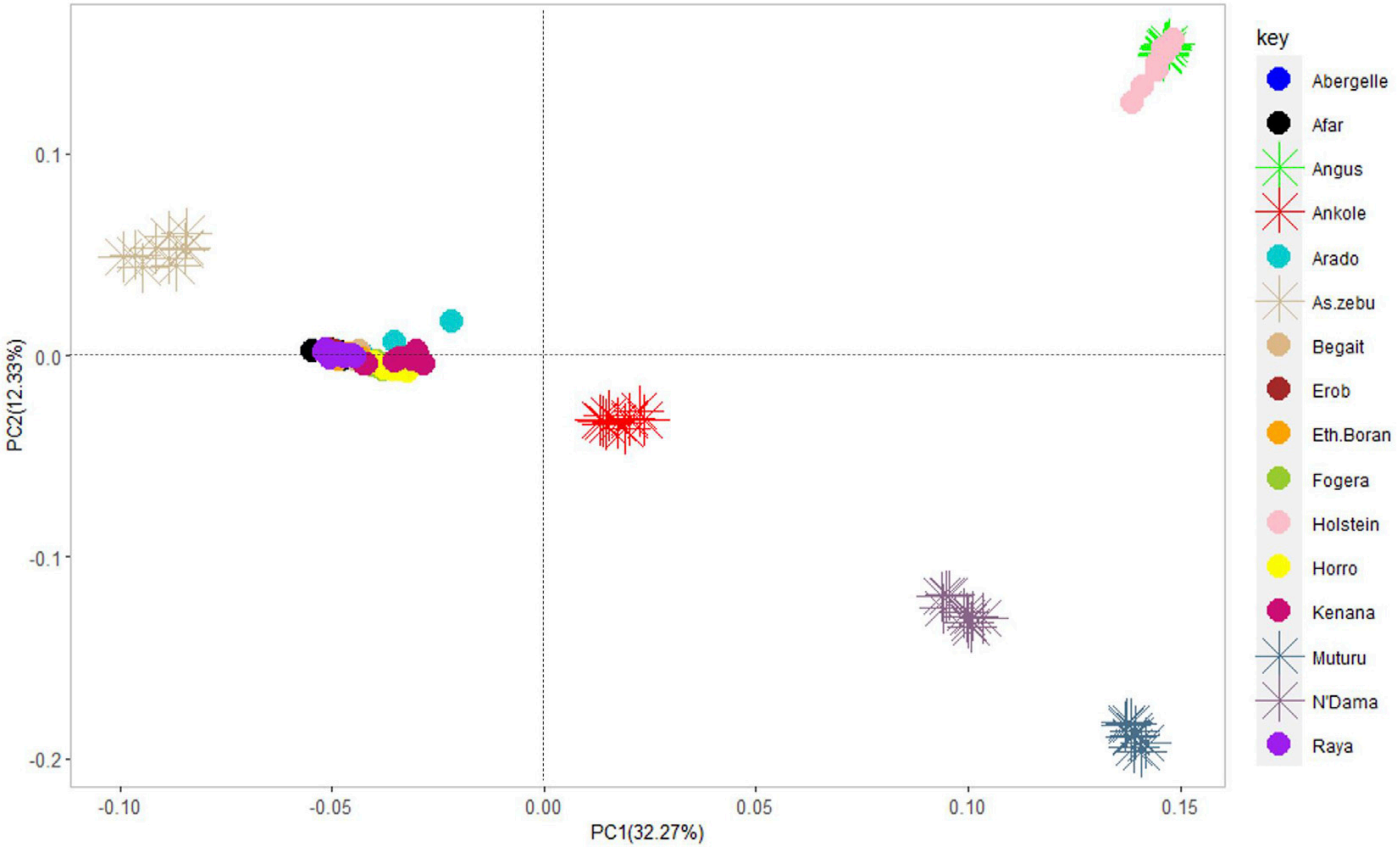
Principal component analysis plot (PC1 and PC2) of the five Tigray cattle populations and reference cattle groups. African sanga (Afar and Ankole, crosses between African zebu and longhorn humpless taurine), African zenga (Fogera and Horro, crosses between African zebu and sanga), African zebu (Ethiopian Boran and Kenana), African taurine (Muturu and N’Dama), European taurine (Angus and Holstein) and Asian zebu (Bhagnari, Cholistani, Dhanni, Sahiwal and Tharparkar).

The PCA showed the presence of six potential clusters of populations (Figure 10). PC1 and PC2 explained 32.27% and 12.33% of the total variation, respectively (Figure 10 and Supplementary Figure S7). PC1 separated Ankole and taurine cattle (African and European) from Asian zebu, Kenana and all the cattle from Ethiopia, including the Tigray cattle (Abergelle, Arado, Begait, Erob and Raya). PC2 divided the European taurine cattle and Asian zebu from the Ankole and African taurine cattle (Figure 10). Combining PC1 and PC2 illustrated that the Tigray cattle populations (Abergelle, Arado, Begait, Erob and Raya) were close to the Asian zebu and the African sanga (Ankole).

**FIGURE 11:**
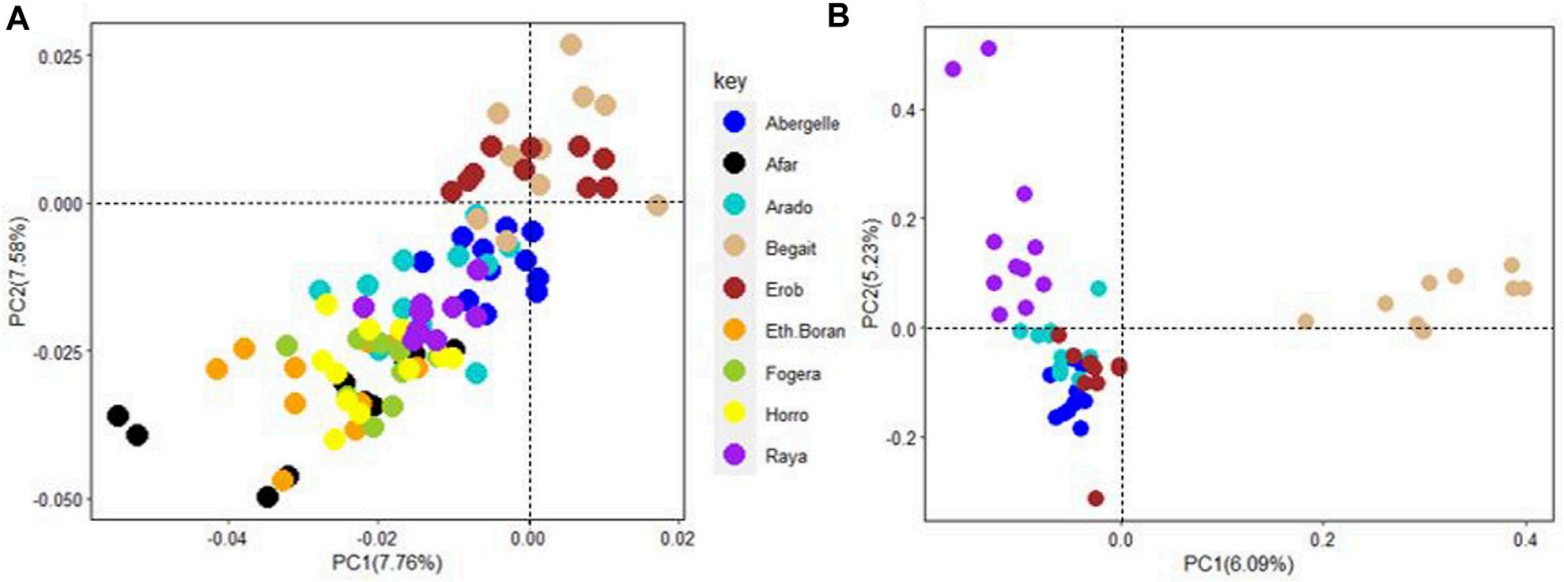
**(A)** Principal component analysis plot (PC1 and PC2) of the five Tigray cattle populations and other Ethiopian cattle representing three cattle groups of African zebu (Ethiopian Boran), African sanga (Afar) and African zenga (Fogera and Horro). **(B)** Principal component analysis plot (PC1 and PC2) for the five Tigray cattle populations alone (Abergelle, Arado, Begait, Erob and Raya).

PC1 and PC2 for the five Tigray cattle populations and other Ethiopian cattle representing three cattle groups of African zebu (Ethiopian Boran), African sanga (Afar) and African zenga (Fogera and Horro) jointly accounted for 15.34% of the total variation, of which the Begait and Erob cattle were separated from the other populations (Figure 11A). The PC1 (6.09%) of the five Tigray cattle populations alone separated Begait cattle from the other four Tigray cattle populations, while PC2 (5.23%) divided Raya cattle from the other four Tigray cattle populations (Figure 11B).

#### Genetic admixture and population genetic differentiation

**FIGURE 12:**
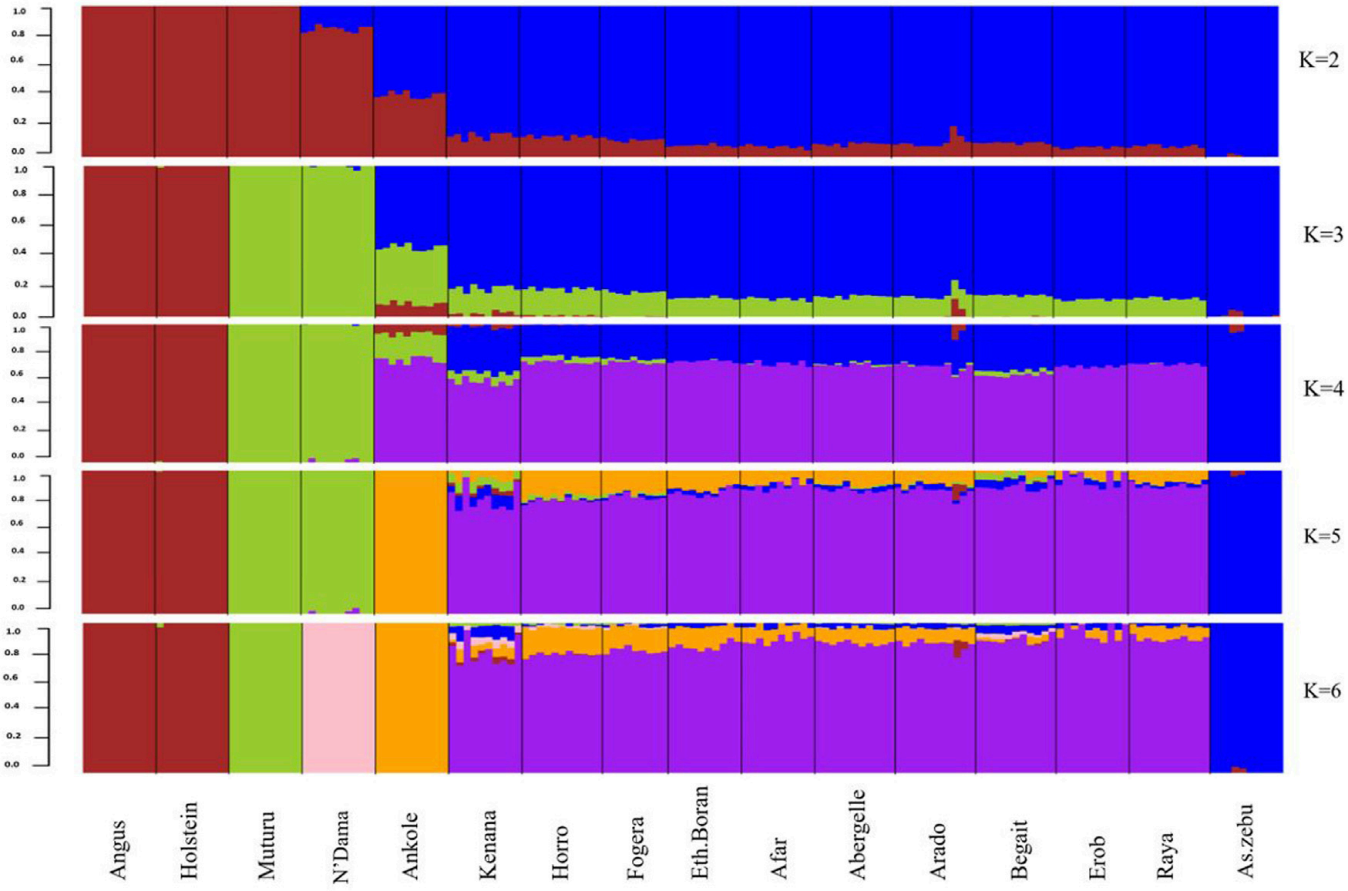
Admixture at K = 2 to K = 6 (the black lines separate the populations labelled below the figure).

As indicated by the lowest cross-validation error (0.51) (Supplementary Figure S8), the admixture analysis suggested three ancestral sources. At K = 3, the taurine ancestry for the Tigray cattle was shown to be mainly shared with the African taurine, except for some individuals in Arado (n = 3) and Begait (n = 2) cattle having 0.1%–1.8% of European taurine ancestry (Figure 12). In each population, the African taurine ancestry ranged from 11.3% (Erob cattle) to 14.1% (Begait cattle) and, accordingly, the indicine ancestry from 85.6% (Arado cattle) to 88.7% (Erob cattle) (Supplementary Figure S9). More interestingly, as the number of potential ancestries increased, the Tigray cattle local ancestry appeared. At K = 7 and K = 10, both Erob and Begait cattle showed some unique local ancestries (Supplementary Figure S10).

**FIGURE 13:**
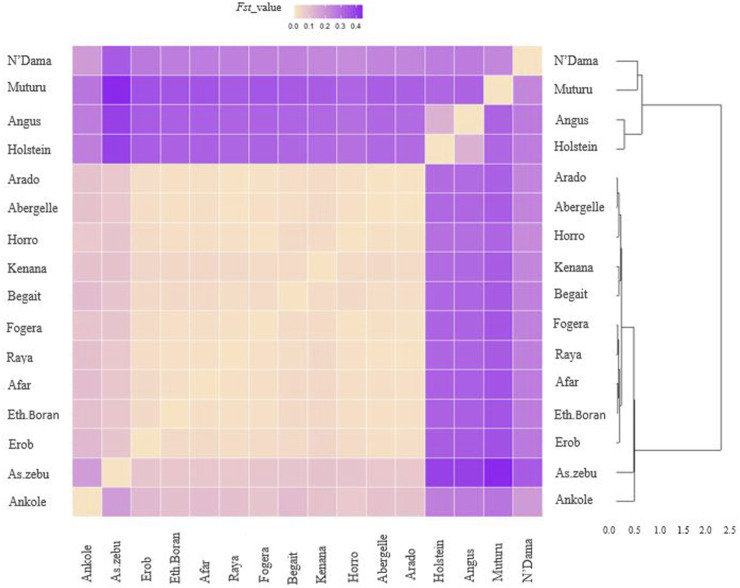
Heat map and dendrogram based on pairwise weighted *F*_ST_ values. The darker colour indicates higher pairwise population differentiation while lighter colour lower population differentiation.

*F*_ST_ for the Tigray cattle populations ranged from 0.07 to 0.08 with Asian Zebu, 0.084 to 0.108 with Ankole (African sanga originating from Uganda), 0.236 to 0.264 with N’Dama (African taurine), 0.328 to 0.36 with Muturu (African taurine) and 0.300 to 0.335 with the European taurine cattle (Angus and Holstein) (Supplementary Table S21). Within Ethiopian cattle, we observed two groups among the Tigray cattle populations for the *F*_ST_ estimates with other Ethiopian cattle populations, with higher genetic differentiation (*F*_ST_ > 0.02) for Begait and Erob cattle from Ethiopian Boran, Fogera and Horro cattle than for Abergelle, Arado and Raya cattle (*F*_ST_ around 0.01) (Figure 13 and Supplementary Table S21).

**FIGURE 14:**
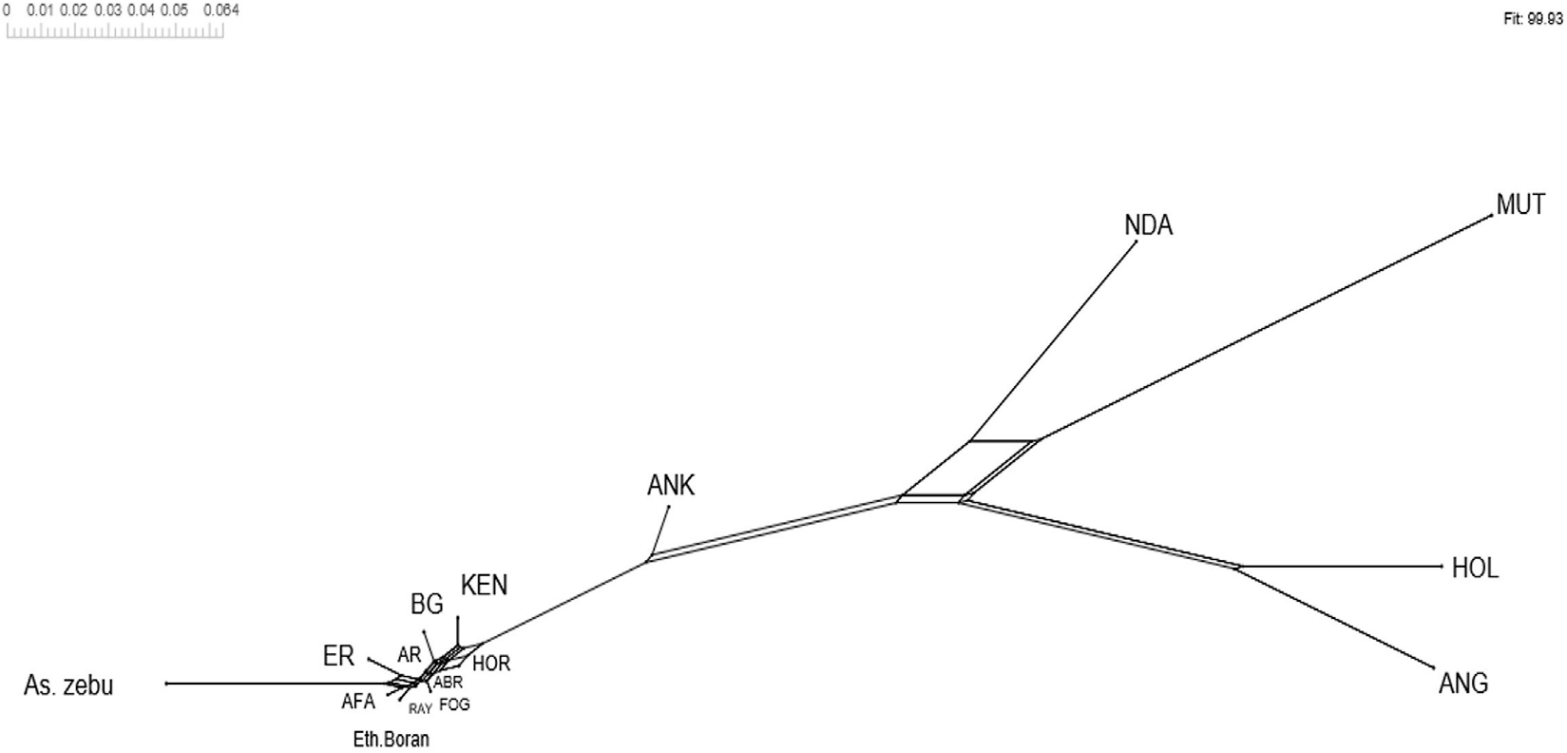
Neighbor-Net tree based on pairwise *F*_ST_ values.

Overall, the heat map and dendrogram (Figure 13 and Supplementary Table S21) generated from pairwise weighted *F*_ST_ values among the Tigray cattle populations and the Tigray cattle populations against other cattle breeds (African sanga, African zenga, African zebu, Asian zebu, African and European taurine cattle) showed two main genetic clades: One comprising the taurine group with two sub-clusters African and European) and another including the Asian zebu and other non-taurine African origin breeds (including the Tigray cattle populations). This was consistent with the PCA and admixture analysis results. Further, the Neighbor-Net tree based on the pairwise *F*_ST_ values (Figure 14) supported the admixture, the heat map and the dendrogram results.

## Discussion

We report the first whole-genome-sequence-based characterisation of the genetic diversity, relatedness and admixture of cattle populations indigenous to the Ethiopia’s Tigray region. We used abundance, distribution and functional description of SNPs and indels, genome-wide nucleotide diversity (*π*), heterozygosity (*Ho*), runs of homozygosity (ROH) and genomic inbreeding coefficient to evaluate the intra-population genetic diversity. The pairwise population differentiation (*F*_ST_) and relationship based on PCA and admixture analysis were employed to assess the inter-population differentiation and relationship among the Tigray cattle and between the Tigray cattle and other reference cattle groups (African sanga, African zenga, African and Asian zebu, and African and European taurine cattle).

We compared our findings with previous studies using the same ARS-UCD1.2 as reference genome (Rosen et al., 2020). For cattle populations as in our study, predominantly of indicine ancestry, this would have likely inflated the number of detected SNPs. Aligning our reads to an indicine reference genome would minimize subspecies ascertainment of SNPs biases (Low et al., 2020).

The alignment rates of the Tigray cattle sequence reads were similar to those of other African (Kim et al., 2020), Chinese (Jiaxian Red, Wenshan, Wannan and Leiqiong) (Zhang et al., 2019; Xia et al., 2021) and European (Angus and Holstein) (Kim et al., 2020) cattle breeds, suggesting the overall similarity in autosomal structures across cattle populations in the world, despite their distinct evolutionary histories.

The high variation in number of genetic variants (SNPs and indels) within and across the Tigray cattle populations illustrated their rich genetic diversity. Also, we found a substantial number of novel variants (SNPs and indels) in the Tigray cattle (Table 1), indicating their importance as a reservoir of genetic diversity previously uncharacterized. Interestingly, we found many new indels (around 34%) compared to novel SNPs. However, it should be emphasized that indels have been given so far less attention in cattle genomic analyses (Stafuzza et al., 2017), despite being part of the important drivers of phenotypic and genetic diversity (Iqbal et al., 2019). Most of the whole genome analyses on African cattle breeds were based on SNPs (Kim et al., 2017; Tijjani et al., 2019; Kim et al., 2020; Jang et al., 2022; Mauki et al., 2022; Terefe et al., 2022; Terefe et al., 2023), while our study is the first to report indels for Ethiopian cattle.

### High genetic “functional” variability in the Tigray cattle

We observed private variants in each Tigray cattle population. Though they only represented around 1.5% (Erob cattle) to 2.1% (Arado cattle) of the total variants in respective populations, they might serve as important diagnostic markers. A small proportion of these SNPs (around 0.23%–0.32%) were missense variants, of which the majority were located in coding regions (97.9%–98.9%), while a few (1.1%–2.1%) were in splice regions. Amongst these SNPs, one-third to two-thirds had a predicted deleterious effect.

Further analysis of all private missense variants identified several GO terms and KEGG pathways shared by different populations or to be population specific. The two most significant GO terms of the molecular function (the olfactory receptor activity and odorant binding) and one most significant KEGG pathway (olfactory transduction) present in the five Tigray cattle populations were associated with olfaction or odour recognition. An efficient olfactory reception is an important fitness mechanisms essential for adaptation, including food and water search behaviour and reproduction (Kour et al., 2022). Odour recognition influences food intake identification and preference (Soria-Gómez et al., 2014).

The GO terms of the cellular component related to the integral component of the plasma membrane (GO:0005887) and its subtype intrinsic component of the plasma membrane (GO:0031226) were only found in Begait cattle. In these GO terms, several important genes were found to be associated with morphology, production, reproduction, feed efficiency, immune response and environmental adaptation. For example, *SCN4A* (Cai et al., 2019) and *TAS1R2* (Zhang et al., 2012) were reported to be associated with body height in cattle. *KCNG4* was found to be related to morphometric traits like rump height, body length and chest depth in goats (Easa et al., 2022). *FLT4* was identified to be relevant to proliferation and growth in cattle (Keogh et al., 2019). *GABRG1* was implicated in milk yield (Pedrosa et al., 2021). Other genes in Begait cattle included *PCDH8* (Taussat et al., 2020) and *SLC26A3* (Kern et al., 2016) associated with feed efficiency in cattle, *PCDH18* related to the immune system and adipogenesis (de Lima et al., 2020). *DUOX2* is important for thyroid hormones production and in innate immunity (Maruo et al., 2016), *Mfsd2b* important in S1P transport activity (Kobayashi et al., 2018) essential for various cellular functions (Spiegel and Milstien, 2011; Cyster and Schwab, 2012), *ITGA5* involved in different inflammation and immune response functions such as PI3K–Akt signaling pathway, bacterial invasion of epithelial cells, phagosome and human papillomavirus infection (Wang et al., 2021). *NPFFR1* (Moulédous et al., 2010) and *HTR7* (Hedlund et al., 2003) are important in body temperature regulation. Last but not least, *Kcnv2* was reported to be associated with visual adaptation in a changing lighting condition environment (Hölter et al., 2012).

Two significant GO terms of the cellular component (GO:0005578∼proteinaceous extracellular matrix and GO:0044420∼extracellular matrix component) were explicitly enriched in Erob cattle. Genes such as multimerin 2 (*MMRN2*), von Willebrand factor C domain containing 2 (*VWC2*) and laminin subunit gamma 1 (*LAMC1*) were important in these GO terms. The *MMRN2* is associated with a meat quality trait called meat juiciness (Leal-Gutiérrez et al., 2019). Similarly, *VWC2* was considered as a candidate gene for intramuscular fat content, one of the most important meat quality traits in beef cattle (Halli et al., 2022). *VWC2* was also reported to be associated to feed efficiency in pigs (Wang et al., 2015). *LAMC1* was involved in different inflammation and immune response pathways, including prion diseases (bovine spongiform encephalopathy), amoebiasis and toxoplasmosis in cattle. Moreover, *LAMC1* was also shown to be relevant to temperature range in cattle (Flori et al., 2019).

Six molecular function GO terms relevant to intra- or extra-cellular activity were significantly enriched in several Tigray cattle populations (Abergelle, Arado, Begait and Raya), in which a few genes such as cadherin EGF LAG seven-pass G-type receptor 1 (*CELSR1*), gamma-aminobutyric acid type A receptor rho3 subunit (*GABRR3*), plexin A2 (*PLXNA2*) and toll-like receptor 6 (*TLR6*) were identified in Abergelle, Arado and Raya cattle, while the gene macrophage stimulating 1 receptor (*MST1R*) was overrepresented in the six GO terms (in Abergelle, Arado, Begait and Raya). *CELSR1* (Guo Y. et al., 2019) was found to be overexpressed following *in vitro* treatment of lipopolysaccharide, a cause of the endometrium inflammation (Sheldon et al., 2010), supporting its importance in immune response. In significantly enriched GO terms in Abergelle, Arado and Raya cattle, we found *TLR6* as an important candidate gene for bovine tuberculosis resistance (Song et al., 2014). Several studies (Zhang et al., 2009; Seabury et al., 2010; Fisher et al., 2011; Elmaghraby et al., 2018; Maurić Maljković et al., 2023) have reported the importance of toll-like receptor genes for immunity, disease resistance and adaptive immune responses, including mastitis, the most economically important disease in dairy cattle (Elmaghraby et al., 2018; Maurić Maljković et al., 2023). Other genes relevant to oxidative stress (*MST1*) (Xiao et al., 2011), cattle temperament (*PLXNA2*) (Gutiérrez-Gil et al., 2008) and fertility such as sperm motility (*GABRR3*) (Hering et al., 2014) were also present in the six GO terms (Abergelle, Arado and Raya cattle).

In our previous morphological study of the same Tigray cattle populations (Zegeye et al., 2021), we showed that four of the five populations may be separated using morphological criteria. The exception was Erob and Abergelle, with a similar morphology. In particular, Begait cattle had the largest body size, a finding in agreement with the missense variants within genes linked to body height and length. Also, the presence of missense variants in genes involved in olfaction may be attributed to the adaptation of the Tigray cattle to the dry agro-ecology in the region, a characteristic of the Sudano-Sahelian ecology with heat and water stress as an issue (Nyssen et al., 2009; Kumasi and Asenso-Okyere, 2011; Abraha, 2013). In addition, the regional landscape is mainly composed of mountains and hills (Kumasi and Asenso-Okyere, 2011) with limited grazing resources. As a result, the Tigray cattle are strongly adapted to feed shortage, as evidenced by the overrepresentation of genes associated with the olfactory and sensory perception of smell to differentiate the edible from non-edible or palatable from non-palatable browse plant species.

### High genetic diversity within and across the Tigray cattle populations

There is a high genome-wide nucleotide diversity (*π*) in all Tigray cattle, comparable with the values observed in Asian indicine cattle but higher than those in taurine cattle (Muturu, N’Dama, Angus and Holstein) (Figure 7) and indicine-taurine admixed (*π* = 2.9 × 10^−3^ for Jiaxian Red) cattle (Xia et al., 2021). Similarly, the observed heterozygosity (*Ho*), an important indicator of genetic variability in domestic animals (Zhang et al., 2018), ranging from 0.278 to 0.302 among the Tigray cattle was similar with other indicine but higher than African and European taurine cattle.

Historic factors associated with the arrival and admixture of cattle in the Horn of Africa, including the Tigray region, likely shaped today’s genome diversity of the Tigray cattle. As an ancient centre of civilisation, the Tigray region probably witnessed the early arrival of taurine cattle, followed by late introductions of indicine cattle in several migration waves, which continuously enriched the genomic landscape of the Tigray cattle. While our results indicated a large proportion of indicine background in Tigray cattle (around 90%), we still found a proportion of African taurine ancestry in their genomes. We may reasonably hypothesise that the rich genetic variation present in modern Tigray cattle is a legacy of multiple introductions, admixture and dispersion of cattle across the Horn of Africa.

We compared the ROH distribution pattern across the Tigray cattle populations and between the Tigray cattle and other reference cattle groups included in this study and found that all the Tigray cattle showed different patterns of ROH as compared to Asian zebu, African taurine (N’Dama and Muturu), African sanga (Ankole), African zebu (Kenana) and European-taurine (Angus and Holstein) cattle. As expected from their high genomic diversity, the number and cumulative length of ROH were smaller in the Tigray cattle compared with previous reports for taurine cattle (Purfield et al., 2012; Xia et al., 2021) and indicine-taurine admixed cattle outside Ethiopia (Xia et al., 2021). However, the number and length of ROH observed in the Tigray cattle were similar to the one reported for other Ethiopian breeds included in our study (Horro, Fogera, Borana, and Afar). It suggests common breeding history among Ethiopian cattle breeds, while PCA and admixture results suggest close genetic relationships among the Ethiopian cattle as recently showed in a genome analysis including 14 Ethiopian indigenous cattle breeds (Terefe et al., 2023).

Inbreeding coefficients were far lower in the Tigray cattle than those reported in other cattle breeds, particularly the Danish dairy cattle breeds (Zhang et al., 2015b), with an inbreeding coefficient five times higher at a population level. An inbreeding coefficient below 5%, as observed for nearly all animals in this study (F_ROH_), is generally considered to have no consequence on an individual’s fitness (Slate et al., 2004). Therefore, the level of inbreeding in the Tigray cattle is within an acceptable range to accommodate within-population improvement of their productivity.

### Population genetic structure and relationship

Taurine ancestry was generally low in the Tigray cattle relative to other African humped cattle breeds (Kim et al., 2020). This is particularly expected for cattle populations geographically close to the entry points of Asian indicine cattle into Africa. The unique local ancestries observed in Erob (K = 7) or Begait (K = 10) and their introgression to all non-taurine African breeds (Supplementary Figure S10) could further confirm the probability of the Tigray region of Ethiopia as a gate of cattle to Africa. Moreover, we observed a closer relationship between Begait to Kenana cattle (a Sudanese cattle breed) than with other Tigray cattle populations. Begait cattle are typically found in the western Tigray regions close to the Sudanese border. Therefore, gene flow from Begait cattle to Sudanese cattle is possible or *vice versa*. Previously, we observed a close morphological relationship between Erob and Abergelle cattle (Zegeye et al., 2021). This result is not supported by our genetic relationship analysis with the two breeds here clearly separated (e.g., *F*_ST_-based dendrogram, Supplementary Figure S11). The two breeds are found at different altitudes (Figure 1B). Henceforth, the relationship between Erob and Abergelle cattle requires further investigation.

## Conclusion

Overall, we provided a detailed analysis using whole genome sequencing data of the genetic diversity, relatedness and admixture of five cattle populations indigenous to the Tigray region, the northernmost state of Ethiopia and a major geographic region of ancient civilizations. We found around 36 M SNPs and 3.7 M indels, where around 7% and 34% of them were novel. The contribution of such novel variants increases the number of known cattle genomic variants and prompts our understanding of the genetic diversity of domestic cattle. We found a high within-population diversity based on the incidence, type and distribution of the genomic variants, genome-wide nucleotide diversity, heterozygosity, runs of homozygosity and genomic inbreeding coefficient. Besides, we detected a sign of poor management in a few Begait and Raya cattle having long ROH and strong inbreeding (>10%), possibly resulting from consanguineous mating. So, these two populations may need special attention to maintain their within-population genetic diversity. The admixture analysis confirmed that the Tigray cattle have a common main indicine ancestry, followed by a low African taurine and a rather limited European taurine ancestry. With high within-population genetic diversity, the Tigray cattle represent an important indigenous genetic resource for breeding improvement to enhance their productivity (e.g., milk), while maintaining their environmental adaptability. All the Tigray cattle populations shared highly significant GO and pathway terms associated with sensory perception of smell with overrepresented genes in the olfactory family, which may be relevant to their adaptation to their harsh environments.

## Data Availability

The datasets presented in this study can be found in online repositories. The names of the repository/repositories and accession number(s) can be found below: NCBI/SRA (https://www.ncbi.nlm.nih.gov/sra). Accession No. PRJNA882591.
