# Supplementary material for: Genome-wide diversity and admixture of five indigenous cattle populations from the Tigray region of northern Ethiopia
Source: Front Genet. 2023 Jul 28;14:1050365. doi: 10.3389/fgene.2023.1050365 (PMC10432725; doi:10.3389/fgene.2023.1050365)
Supplement: Supplementary file 2 [file DataSheet1.docx]

**Supplementary figures**


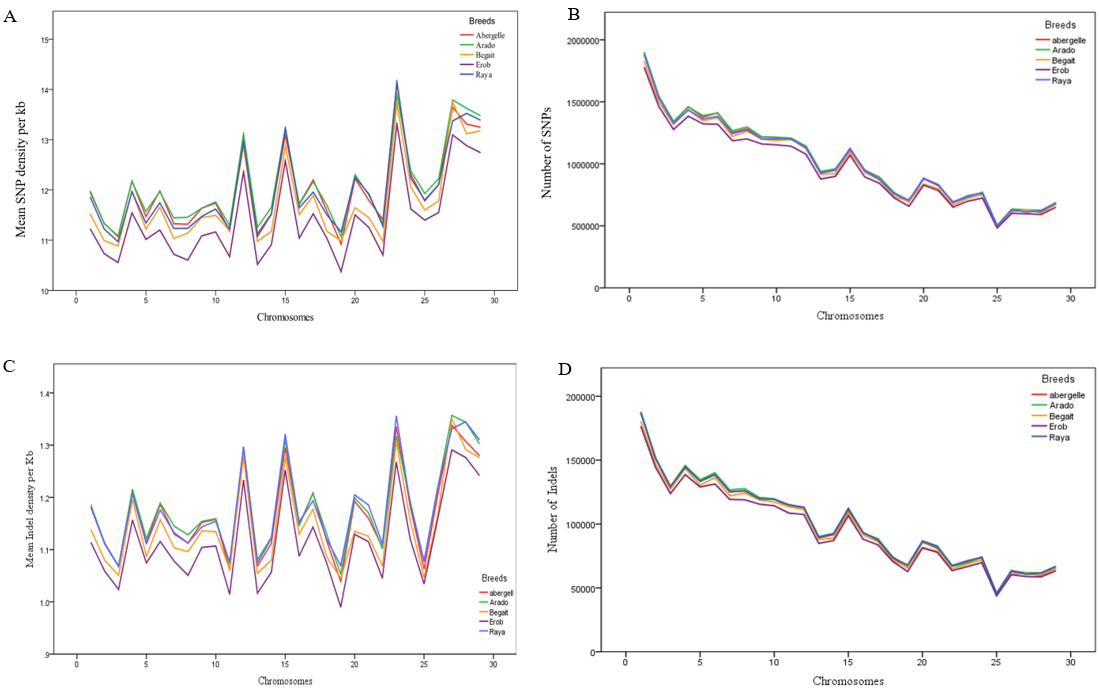
Figure S1. Mean variants density across 1 kb window for Tigray cattle chromosomes. (A) Mean SNPs density (B) mean indel density, (C) SNPs distribution across the chromosomes and (D) Indels SNPs distribution across the chromosomes.


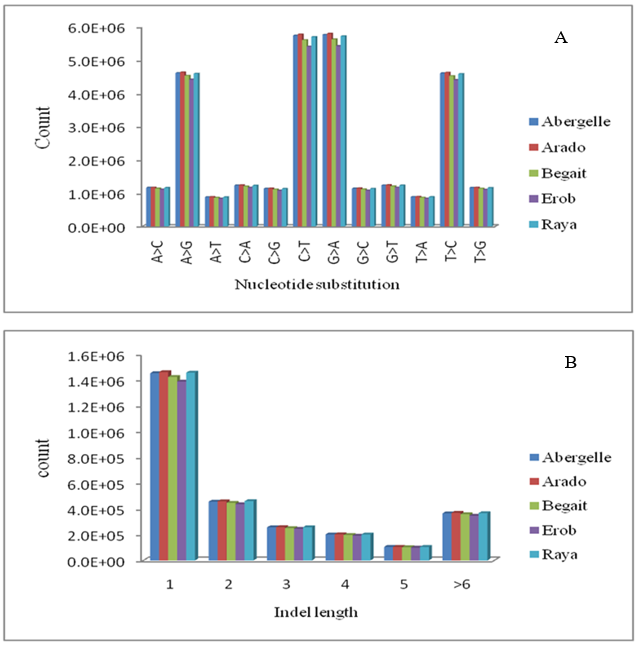


Figure S2. (A) Status of nucleotide substitution (A > C = substitution of Adenine by Cytosine, A > G = Adenine by Guanine, A > T = Adenine by Thymine, C > A = Cytosine by Adenine, C > G = Cytosine by Guanine, C > T = Cytosine by Thymine, G > A = Guanine by Adenine, G > C = Guanine by Cytosine G > T = Guanine by Thymine, T > A = Thymine by Adenine, T > C = Thymine by cytosine and T > G = Thymine by Guanine) across the Tigray cattle populations and (B) Distribution of indel length across the genome of Tigray cattle.


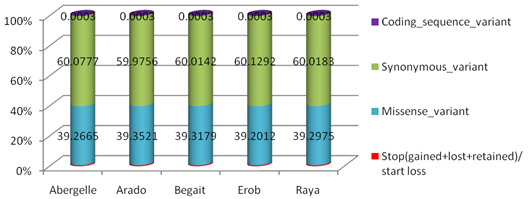


Figure S3. Showing the polymorphism consequences.


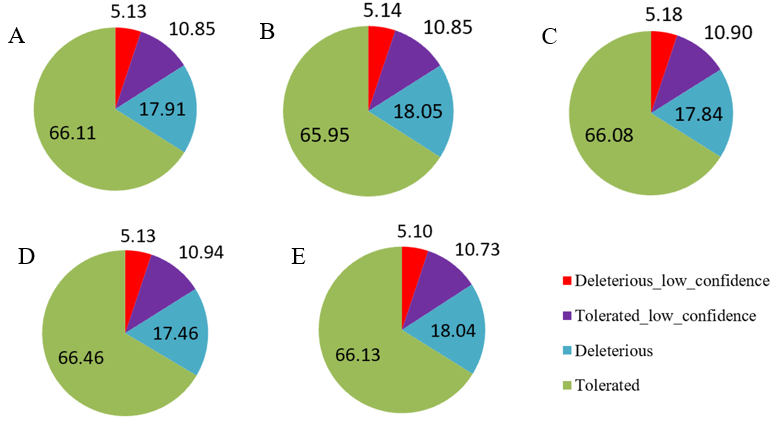


Figure S4. VEP based SIFT analysis for amino acid altering SNPs (%). (A) Abergelle, (B) Arado, (C), Begait, (D) Erob and (E) Raya.


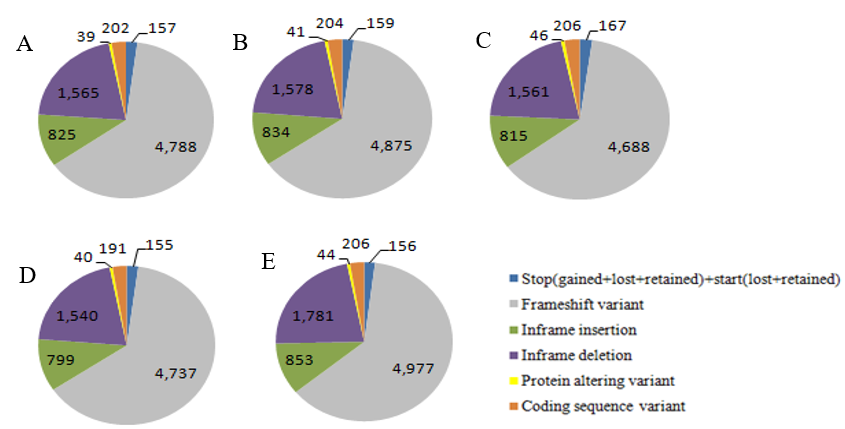


Figure S5. Indels coding consequence. (A) Abergelle (B) Arado (C) Begait (D) Erob and (E) Raya.


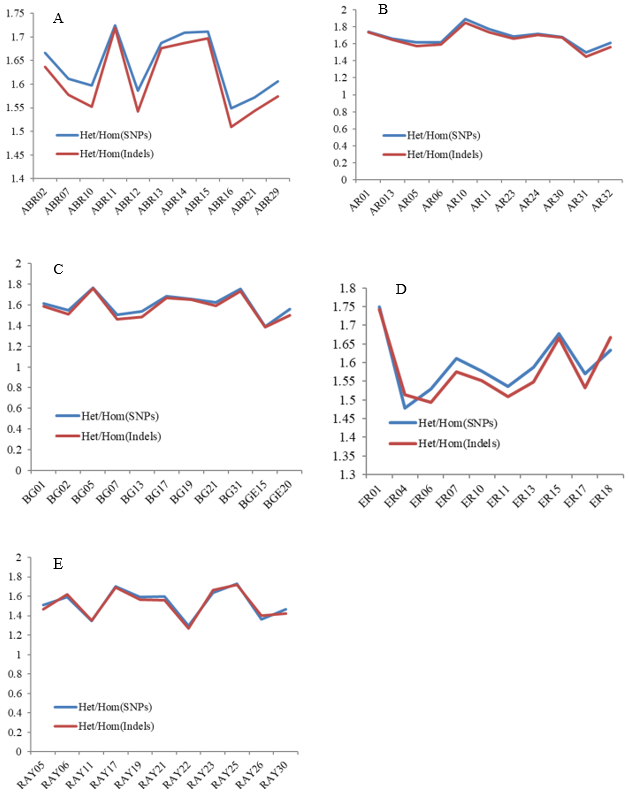


Figure S6. Distribution of heterozygous-to-homozygous variants among individual samples. (A) Abergelle (B) Arado, (C) Begait, (D) Erob and (E) Raya.


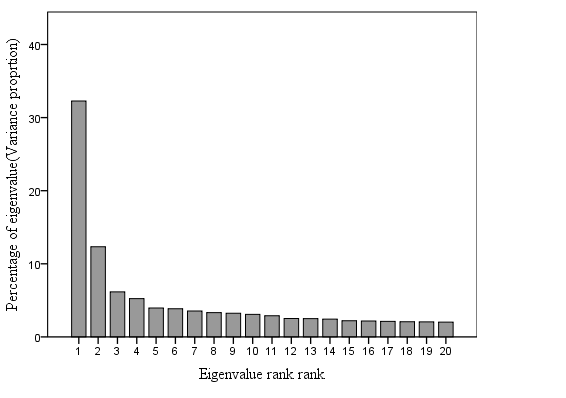


Figure S7. Proportion of variance explained by each principal component (1 to 20).


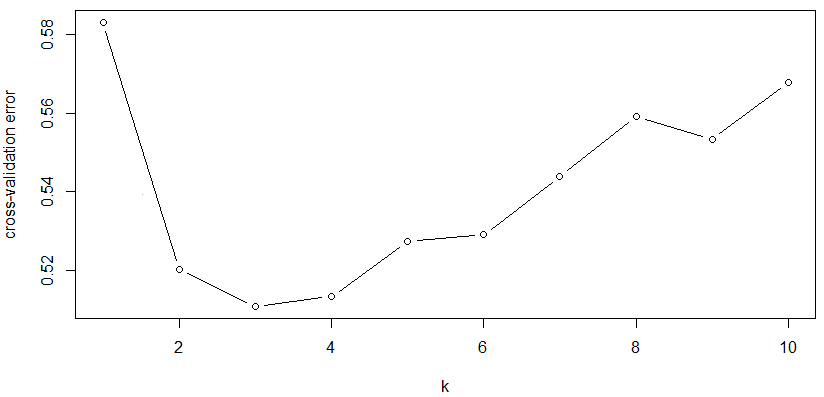


Figure S8. Cross-validation errors of different k values used for admixture analysis.


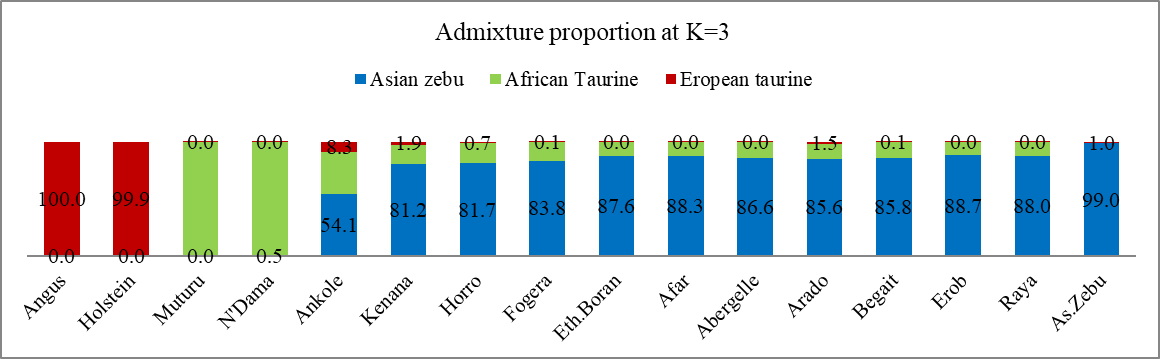


Figure S9. Indicine and taurine ancestry proportion (%) at K = 3 based on the Q (ancestry fractions) = 3.


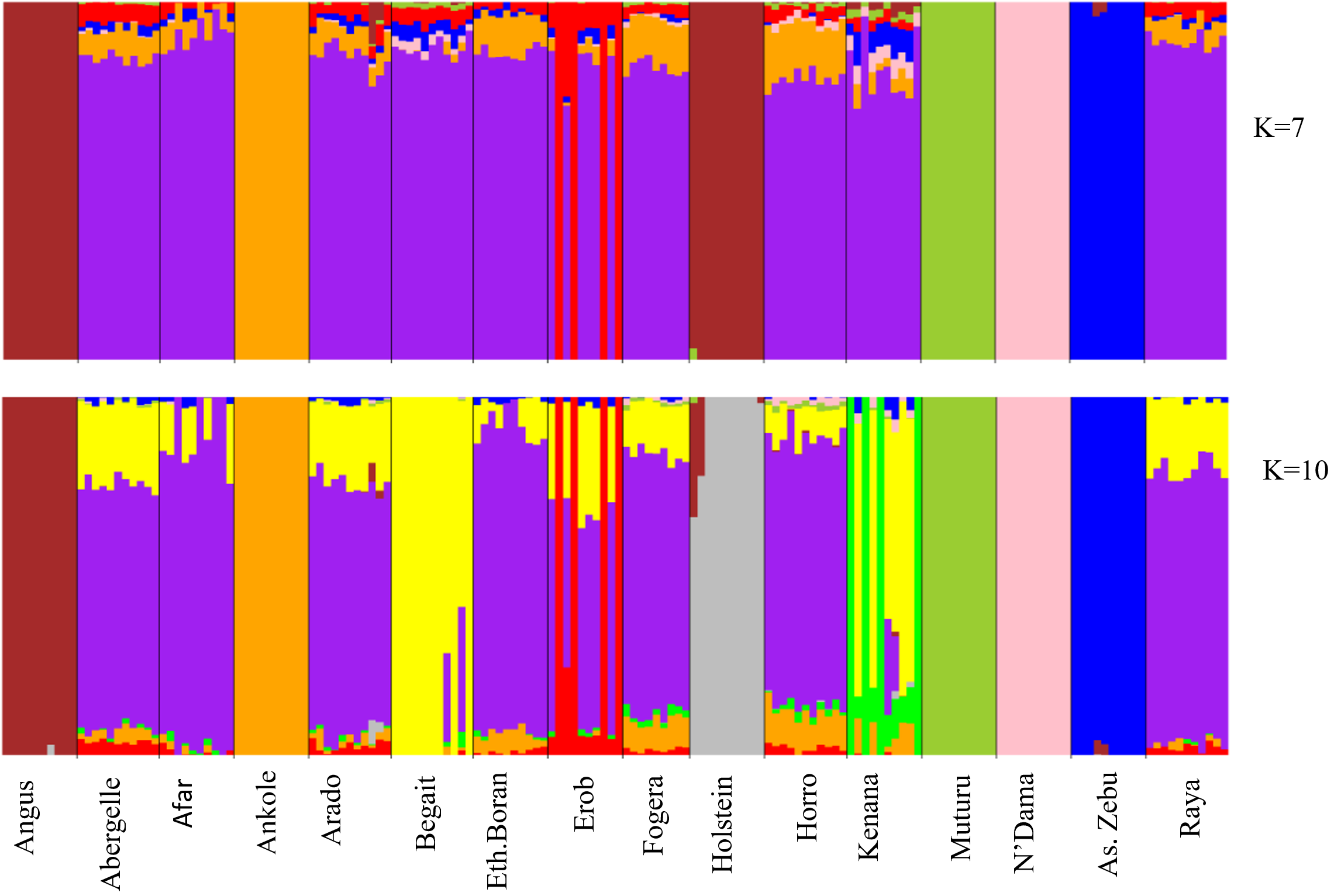


Figure S10. Admixture k = 7 and k = 10 showing unique local ancestry for Erob and Begait cattle, respectively (the colors represent each K population cluster and black lines separate the populations labeled below the figure).

**Supplementary tables**

Table S1. Extracted gDNA quality reports.

| Sample^1^ | Sex | Population | Concentration in ng/uL | 260/280 | 260/230 |
| --- | --- | --- | --- | --- | --- |
| ABR02 | F | Abergelle | 65.729 | 1.8 | 2.28 |
| ABR07 | F | Abergelle | 127.176 | 1.8 | 1.93 |
| ABR10 | M | Abergelle | 105.025 | 1.75 | 2.16 |
| ABR11 | M | Abergelle | 219.777 | 1.81 | 1.81 |
| ABR12 | F | Abergelle | 50.37 | 1.75 | 1.92 |
| ABR13 | M | Abergelle | 53.963 | 1.77 | 1.79 |
| ABR14 | F | Abergelle | 61.672 | 1.76 | 1.84 |
| ABR15 | F | Abergelle | 51.138 | 1.77 | 1.65 |
| ABR16 | M | Abergelle | 55.075 | 1.89 | 2.19 |
| ABR21 | M | Abergelle | 61.815 | 1.84 | 1.9 |
| ABR29 | F | Abergelle | 62.386 | 1.78 | 1.77 |
| AR01 | F | Arado | 55.65 | 1.77 | 1.8 |
| AR05 | F | Arado | 53.691 | 1.83 | 1.83 |
| AR06 | F | Arado | 74.482 | 1.74 | 2.05 |
| AR10 | M | Arado | 62.199 | 1.85 | 2.37 |
| AR11 | M | Arado | 60.315 | 1.81 | 1.96 |
| AR13 | F | Arado | 89.223 | 1.83 | 2.05 |
| AR23 | F | Arado | 86.414 | 1.75 | 2.15 |
| AR24 | F | Arado | 83.777 | 1.77 | 1.97 |
| AR30 | F | Arado | 71.169 | 1.79 | 2.1 |
| AR31 | F | Arado | 51.697 | 1.78 | 1.87 |
| AR32 | M | Arado | 66.633 | 1.81 | 1.97 |
| BG01 | F | Begait | 59.349 | 1.75 | 2.19 |
| BG02 | F | Begait | 58.854 | 1.75 | 1.98 |
| BG05 | F | Begait | 54.813 | 1.79 | 1.74 |
| BG07 | F | Begait | 51.566 | 1.77 | 1.75 |
| BG13 | F | Begait | 98.864 | 1.77 | 2.07 |
| BG15 | M | Begait | 157.242 | 1.8 | 2.06 |
| BG17 | F | Begait | 58.33 | 1.77 | 1.99 |
| BG19 | F | Begait | 59 | 1.7 | 2.02 |
| BG20 | F | Begait | 59.508 | 1.76 | 2.11 |
| BG21 | F | Begait | 171.389 | 1.79 | 1.9 |
| BG31 | F | Begait | 78.96 | 1.78 | 2.11 |
| ER01 | M | Erob | 92.316 | 1.78 | 2.11 |
| ER04 | F | Erob | 73.308 | 1.77 | 2.02 |
| ER06 | M | Erob | 174.752 | 1.83 | 2 |
| ER07 | M | Erob | 64.94 | 1.81 | 2.48 |
| ER10 | F | Erob | 100.854 | 1.87 | 2.29 |
| ER11 | F | Erob | 330.196 | 1.74 | 1.94 |
| ER13 | F | Erob | 209.48 | 1.8 | 2.04 |
| ER15 | F | Erob | 96.342 | 1.82 | 1.94 |
| ER17 | M | Erob | 417.424 | 1.81 | 1.95 |
| ER18 | F | Erob | 249.471 | 1.85 | 2.04 |
| RAY05 | M | Raya | 55.058 | 1.73 | 2.07 |
| RAY06 | M | Raya | 53.533 | 1.77 | 2.03 |
| RAY11 | F | Raya | 101.287 | 1.84 | 2.44 |
| RAY17 | F | Raya | 337.08 | 1.83 | 2.14 |
| RAY19 | F | Raya | 59.713 | 1.79 | 1.93 |
| RAY21 | F | Raya | 52.713 | 1.73 | 2.07 |
| RAY22 | F | Raya | 116.279 | 1.85 | 1.84 |
| RAY23 | F | Raya | 62.256 | 1.75 | 2.12 |
| RAY25 | F | Raya | 53.595 | 1.76 | 2.05 |
| RAY26 | F | Raya | 87.681 | 1.78 | 1.83 |
| RAY30 | F | Raya | 57.642 | 1.75 | 2.01 |

^1^*ABR** Abergelle, *RA** Arado cattle, *BG ** Begait, *ER** Erob, and *RAY ** Raya cattle.

Table S2. Description and source of publicly available reference population data on 110 individuals across 16 cattle breeds.

| Breed | Acronym | Land of origin | Number | Bio-project accession numbers |
| --- | --- | --- | --- | --- |
| Afar | AFR | Ethiopia | 10 | PRJNA574857 |
| Angus | AAN | Canada | 10 | PRJNA574857 |
| Ankole | ANK | Uganda | 10 | PRJNA176557 |
| Bhagnari (Asian zebu) | TBG | Pakistan | 2 | PRJNA312138 |
| Cholistani (Asian zebu) | CHO | Pakistan | 2 | CNP0000189 |
| Dhanni (Asian zebu) | DHA | Pakistan | 2 | CNP0000189 |
| Eth.Boran | BOR | Ethiopia | 10 | CNP0000189 |
| Fogera | FOG | Ethiopia | 9 | PRJNA574857 |
| Holstein | HOL | Canada | 10 | PRJNA574857 |
| Horro | HOR | Ethiopia | 11 | PRJNA176557 |
| Kenana | KEN | Sudan | 10 | PRJNA574857 |
| Muturu | MUT | Nigeria | 10 | PRJNA312138 |
| N’Dama | NDA | Guinea | 10 | PRJNA386202 |
| Sahiwal (Asian zebu) | SHW | Pakistan | 2 | PRJNA312138 |
| Tharparkar (Asian zebu) | THP | Pakistan | 2 | CNP0000189 |
| Total sample |  |  | 110 |  |

Table S3. Summary results on sequencing and mapping of reads.

| Sample^1^ | Total reads | MR (%)^2^ | PPR^3^ | MD (X)^4^ | 5_b (%)^5^ | 10_b (%)^6^ | 20_b (%)^7^ | 40_b (%)^8^ |
| --- | --- | --- | --- | --- | --- | --- | --- | --- |
| ABR02 | 223701820 | 99.78 | 97.7 | 10.13 | 88.8 | 38.2 | 1.1 | 0.4 |
| ABR 07 | 225383302 | 99.78 | 97.39 | 9.98 | 85.9 | 35.2 | 1.1 | 0.4 |
| ABR 10 | 211853306 | 99.74 | 97.7 | 9.51 | 84.6 | 31.6 | 0.9 | 0.3 |
| ABR 11 | 265608575 | 99.78 | 97.54 | 11.66 | 92.4 | 52.9 | 1.7 | 0.4 |
| ABR 12 | 206914185 | 99.77 | 97.64 | 9.22 | 83.3 | 29.0 | 0.9 | 0.3 |
| ABR 13 | 267674295 | 99.76 | 97.61 | 11.65 | 91.9 | 51.4 | 1.8 | 0.5 |
| ABR 14 | 258784661 | 99.78 | 97.46 | 11.31 | 91.9 | 50.0 | 1.5 | 0.4 |
| ABR 15 | 254717248 | 99.77 | 97.66 | 11.44 | 90.9 | 50.8 | 1.7 | 0.4 |
| ABR 16 | 219354788 | 99.77 | 97.6 | 9.74 | 85.4 | 33.2 | 0.9 | 0.4 |
| ABR 21 | 224258471 | 99.75 | 97.61 | 10.06 | 88.5 | 38.2 | 1.1 | 0.4 |
| ABR 29 | 215860301 | 99.74 | 97.6 | 9.72 | 87.1 | 33.8 | 1.0 | 0.4 |
| **Average** | **234010087** | **99.77** | **97.59** | **10.40** | **88.25** | **40.39** | **1.25** | **0.39** |
| AR01 | 268732515 | 99.72 | 97.52 | 11.74 | 92.3 | 52.7 | 1.8 | 0.5 |
| AR05 | 207962334 | 99.77 | 97.6 | 9.45 | 86.2 | 31.5 | 0.9 | 0.4 |
| AR 06 | 225872374 | 99.78 | 97.57 | 10.02 | 88.3 | 37 | 1 | 0.4 |
| AR 10 | 228945045 | 99.73 | 97.61 | 10.19 | 88.1 | 38.9 | 1.0 | 0.3 |
| AR 11 | 242935153 | 99.61 | 97.39 | 10.69 | 89.1 | 42.6 | 1.2 | 0.4 |
| AR 13 | 272318578 | 99.77 | 97.73 | 11.94 | 92.4 | 53.9 | 1.9 | 0.5 |
| AR 23 | 236874813 | 99.74 | 97.53 | 10.67 | 89.7 | 42.4 | 1.3 | 0.4 |
| AR 24 | 269700129 | 99.74 | 97.6 | 11.56 | 92.3 | 52.4 | 1.7 | 0.4 |
| AR 30 | 260181861 | 99.74 | 97.57 | 11.46 | 91.3 | 51.9 | 1.8 | 0.4 |
| AR31 | 200684387 | 99.73 | 97.64 | 9.12 | 84.1 | 27.8 | 0.8 | 0.4 |
| AR32 | 207542739 | 99.79 | 97.60 | 9.40 | 84.6 | 31.0 | 0.9 | 0.4 |
| **Average** | **238340903** | **99.74** | **97.58** | **10.57** | **88.95** | **42.01** | **1.30** | **0.41** |
| BG 01 | 241159107 | 99.76 | 97.71 | 10.64 | 89.9 | 43 | 1.3 | 0.4 |
| BG 02 | 224954591 | 99.79 | 97.73 | 9.97 | 87.4 | 35.4 | 1.0 | 0.4 |
| BG 05 | 289752799 | 99.72 | 97.53 | 12.29 | 93.3 | 58.1 | 2.2 | 0.5 |
| BG 07 | 207478671 | 99.74 | 97.69 | 9.26 | 85.0 | 29.6 | 0.8 | 0.3 |
| BG 13 | 220949139 | 99.77 | 97.53 | 9.98 | 86.5 | 34.7 | 1.0 | 0.4 |
| BG 15 | 238733623 | 99.74 | 97.76 | 10.57 | 88.8 | 42.7 | 1.2 | 0.4 |
| BG 17 | 266767650 | 99.69 | 97.53 | 11.37 | 91.4 | 49.0 | 1.7 | 0.5 |
| BG 19 | 279123320 | 99.72 | 97.43 | 12.02 | 92.7 | 55.2 | 2.1 | 0.5 |
| BG 20 | 216099482 | 99.68 | 97.55 | 9.70 | 86.1 | 32.8 | 0.9 | 0.4 |
| BG 21 | 223640202 | 99.77 | 97.7 | 9.91 | 87.6 | 35.6 | 1.0 | 0.4 |
| BG 31 | 248420836 | 99.72 | 97.77 | 11.28 | 91.8 | 49.6 | 1.5 | 0.4 |
| **Average** | **241552675** | **99.74** | **97.63** | **10.64** | **89.14** | **42.34** | **1.34** | **0.42** |
| ER 01 | 282881666 | 99.71 | 97.43 | 12.27 | 92.3 | 57.3 | 2.3 | 0.4 |
| ER04 | 200721988 | 99.67 | 97.55 | 9.30 | 85.8 | 30.9 | 0.9 | 0.4 |
| ER 06 | 212690117 | 99.73 | 97.73 | 9.53 | 86 | 31.8 | 0.9 | 0.4 |
| ER 07 | 226350254 | 99.68 | 97.19 | 10.14 | 87.2 | 38.3 | 1.1 | 0.4 |
| ER 10 | 227694454 | 99.76 | 97.72 | 10.17 | 88.6 | 38.1 | 1.1 | 0.4 |
| ER 11 | 224425333 | 99.75 | 97.51 | 9.93 | 88.0 | 36.7 | 1.1 | 0.4 |
| ER13 | 215739659 | 99.71 | 97.27 | 9.83 | 86.6 | 35.7 | 1.0 | 0.4 |
| ER 15 | 260329447 | 99.74 | 97.58 | 11.65 | 92.3 | 53.0 | 1.8 | 0.4 |
| ER 17 | 212074051 | 99.77 | 97.43 | 9.56 | 85.2 | 32.4 | 0.9 | 0.4 |
| ER 18 | 202140293 | 99.75 | 97.58 | 8.88 | 82.4 | 25.7 | 0.8 | 0.3 |
| **Average** | **226504726** | **99.73** | **97.50** | **10.13** | **87.44** | **37.99** | **1.19** | **0.39** |
| RAY 05 | 205800875 | 99.74 | 97.55 | 9.19 | 82.3 | 27.9 | 0.8 | 0.3 |
| RAY 06 | 201381053 | 99.77 | 97.43 | 9.09 | 82.3 | 28.1 | 0.9 | 0.3 |
| RAY11 | 288609469 | 99.71 | 97.44 | 12.69 | 93.7 | 61.9 | 2.7 | 0.5 |
| RAY 17 | 251020395 | 99.74 | 97.66 | 11.13 | 91.3 | 47.9 | 1.5 | 0.4 |
| RAY 19 | 235906893 | 99.77 | 97.45 | 12.02 | 92.7 | 55.2 | 2.1 | 0.5 |
| RAY 21 | 218822571 | 99.78 | 97.57 | 9.86 | 87.6 | 35.6 | 1.0 | 0.4 |
| RAY 22 | 213692973 | 99.69 | 97.41 | 9.7 | 87.2 | 34.5 | 1.0 | 0.4 |
| RAY 23 | 203433032 | 99.71 | 97.53 | 9.20 | 83.9 | 28.5 | 0.9 | 0.4 |
| RAY 25 | 276489917 | 99.74 | 97.62 | 11.83 | 92.6 | 54.9 | 2.0 | 0.5 |
| RAY 26 | 201246838 | 99.71 | 97.72 | 9.12 | 84.6 | 28.7 | 0.8 | 0.3 |
| RAY 30 | 203685341 | 99.74 | 97.51 | 9.26 | 84.7 | 29.6 | 0.9 | 0.4 |
| **Average** | **208571680** | **90.67** | **97.54** | **10.28** | **87.54** | **39.35** | **1.33** | **0.40** |

^1^*ABR ** Abergelle, *RA** Arado cattle, *BG ** Begait, *ER ** Erob, and *RAY ** Raya

^2^*MR* Total numbers of reads mapped to the *ARS-UCD1.2* reference genome.

^3^*PPR*Percentage of both mates of a read pair that were properly mapped to the same chromosome.

4*MD* Mean sequence depth

^5^*5_b*Percentage of the genome with bases covered by at least 5 reads.

^6^*10_b* Percentage of the genome with bases covered by at least 10 reads.

^7^*20_b* Percentage of the genome with bases covered by at least 20 reads.

^8^*40_b* Percentage of the genome with bases covered by at least 40 reads.

Table S4A. Numerical description of variants statistics within individual samples of the Tigray cattle.

| Abergelle | | | Arado | | | Begait | | | Erob | | | Raya | | |
| --- | --- | --- | --- | --- | --- | --- | --- | --- | --- | --- | --- | --- | --- | --- |
| SID | SNPs | Indels | SID | SNPs | Indels | SID | SNPs | Indels | SID | SNPs | Indels | SID | SNPs | Indels |
| ABR02 | 13058773 | 1188687 | AR01 | 13455910 | 1231918 | BG01 | 13091236 | 1194836 | ER01 | 13662412 | 1251926 | RAY05 | 12787007 | 1158602 |
| ABR07 | 12960882 | 1174721 | AR05 | 12812530 | 1161847 | BG02 | 12892820 | 1170639 | ER04 | 13308307 | 1225010 | RAY06 | 13542464 | 1245557 |
| ABR10 | 12766726 | 1154975 | AR06 | 13033024 | 1185364 | BG05 | 13412787 | 1231333 | ER06 | 12979295 | 1178184 | RAY11 | 13088729 | 1204596 |
| ABR11 | 13442658 | 1232531 | AR10 | 12439525 | 1135780 | BG07 | 12666214 | 1144657 | ER07 | 13226134 | 1199475 | RAY17 | 13330632 | 1218156 |
| ABR12 | 12819542 | 1162114 | AR11 | 12931734 | 1180923 | BG13 | 12879773 | 1164489 | ER10 | 13168691 | 1199070 | RAY19 | 13089778 | 1191972 |
| ABR13 | 13440482 | 1228474 | AR13 | 13489412 | 1232186 | BG15 | 12891752 | 1177252 | ER11 | 13145366 | 1195947 | RAY21 | 12913445 | 1173112 |
| ABR14 | 13243509 | 1210777 | AR23 | 13260526 | 1206400 | BG17 | 13180771 | 1204943 | ER13 | 13055806 | 1184993 | RAY22 | 12519807 | 1139075 |
| ABR15 | 13307556 | 1218162 | AR24 | 13466640 | 1232561 | BG19 | 13413410 | 1228804 | ER15 | 13489909 | 1232265 | RAY23 | 13576365 | 1246932 |
| ABR16 | 12946145 | 1176254 | AR30 | 13468688 | 1234899 | BG20 | 12745009 | 1148088 | ER17 | 13000399 | 1181237 | RAY25 | 13495839 | 1235819 |
| ABR21 | 13158414 | 1195051 | AR31 | 12540490 | 1129855 | BG21 | 12943679 | 1175761 | ER18 | 13543563 | 1246164 | RAY26 | 13152030 | 1211620 |
| ABR29 | 12839547 | 1165276 | AR32 | 12814441 | 1160965 | BG31 | 13277275 | 1210669 |  |  |  | RAY30 | 12763148 | 1152548 |

*SID* Sample ID, *ABR** Abergelle, *RA** Arado cattle, *BG** Begait, *ER** Erob, and *RAY** Raya.

Table S4B. Numerical description of variants statistics across the Tigray cattle breeds.

| Variants | Populations | | | | | |
| --- | --- | --- | --- | --- | --- | --- |
|  | ABR (N = 11) | AR (N = 11) | BG (N = 11) | ER (N = 10) | RAY (N = 11) | TCP (N = 54) |
| SNPs |  |  |  |  |  | 36003573 |
| Shared | 2113093 (7.15%) | 2062642 (6.94%) | 2182704 (7.54%) | 2724442 (9.71%) | 2161735 (7.35%) | 674,019 (1.87%) |
| Indels |  |  |  |  |  | 3703659 |
| Shared | 177353 (9.32%) | 173842 (9.08%) | 183302 (9.85) | 228704 (12.59%) | 181610 (9.58%) | 1.43% (52,992) |

*TCP* Tigray cattle population (including all the five populations with 54 animals).

*ABR* Abergelle, *AR* Arado, *BG* Begait, *ER* Erob, *RAY* Raya

Table S5. SNP density per kb across each chromosome (Mean ± SD).

| Chromosomes | ABR (N = 11) | AR (N = 11) | BG (N = 11) | ER (N = 10) | RAY (N = 11) |
| --- | --- | --- | --- | --- | --- |
| 1 | 11.956 ± 7.46 | 11.988 ± 7.46 | 11.522 ± 7.35 | 11.231 ± 7.25 | 11.863 ± 7.44 |
| 2 | 11.34 ± 7.02 | 11.333 ± 7.04 | 10.994 ± 6.94 | 10.735 ± 6.85 | 11.227 ± 7.03 |
| 3 | 11.07 ± 7.29 | 11.088 ± 7.28 | 10.884 ± 7.21 | 10.556 ± 7.15 | 10.971 ± 7.28 |
| 4 | 12.179 ± 8.62 | 12.166 ± 8.56 | 11.972 ± 8.54 | 11.545 ± 8.4 | 11.965 ± 8.57 |
| 5 | 11.47 ± 7.94 | 11.575 ± 7.94 | 11.22 ± 7.93 | 11.016 ± 7.86 | 11.341 ± 7.94 |
| 6 | 11.984 ± 7.14 | 11.965 ± 7.16 | 11.654 ± 7.09 | 11.203 ± 7.02 | 11.744 ± 7.13 |
| 7 | 11.328 ± 7.81 | 11.444 ± 7.92 | 11.034 ± 7.76 | 10.722 ± 7.63 | 11.237 ± 7.83 |
| 8 | 11.318 ± 7.06 | 11.456 ± 7.08 | 11.141 ± 6.99 | 10.604 ± 6.83 | 11.236 ± 7.05 |
| 9 | 11.636 ± 7.42 | 11.637 ± 7.42 | 11.45 ± 7.29 | 11.086 ± 7.22 | 11.472 ± 7.37 |
| 10 | 11.738 ± 8.53 | 11.763 ± 8.52 | 11.495 ± 8.42 | 11.165 ± 8.34 | 11.621 ± 8.5 |
| 11 | 11.18 ± 7.05 | 11.294 ± 7.08 | 11.196 ± 7.04 | 10.673 ± 6.9 | 11.221 ± 7.06 |
| 12 | 13.113 ± 9.43 | 13.099 ± 9.44 | 12.876 ± 9.18 | 12.361 ± 9.27 | 12.958 ± 9.2 |
| 13 | 11.128 ± 7.24 | 11.257 ± 7.24 | 10.976 ± 7.18 | 10.518 ± 7.01 | 11.073 ± 7.19 |
| 14 | 11.528 ± 7.13 | 11.655 ± 7.2 | 11.177 ± 7.01 | 10.913 ± 6.99 | 11.517 ± 7.16 |
| 15 | 13.089 ± 9.44 | 13.228 ± 9.47 | 12.868 ± 9.45 | 12.581 ± 9.18 | 13.231 ± 9.48 |
| 16 | 11.722 ± 7.52 | 11.709 ± 7.53 | 11.508 ± 7.42 | 11.042 ± 7.25 | 11.644 ± 7.52 |
| 17 | 12.202 ± 7.58 | 12.165 ± 7.55 | 11.888 ± 7.44 | 11.528 ± 7.41 | 11.958 ± 7.48 |
| 18 | 11.568 ± 8.41 | 11.684 ± 8.56 | 11.172 ± 8.34 | 11.027 ± 8.33 | 11.495 ± 8.49 |
| 19 | 10.921 ± 7.5 | 11.069 ± 7.54 | 10.986 ± 7.47 | 10.376 ± 7.25 | 11.168 ± 7.55 |
| 20 | 12.226 ± 7.45 | 12.303 ± 7.46 | 11.647 ± 7.29 | 11.505 ± 7.24 | 12.253 ± 7.49 |
| 21 | 11.79 ± 7.8 | 11.892 ± 7.76 | 11.45 ± 7.78 | 11.252 ± 7.62 | 11.919 ± 7.93 |
| 22 | 11.412 ± 7.18 | 11.33 ± 7.2 | 10.974 ± 7.06 | 10.705 ± 7.01 | 11.277 ± 7.14 |
| 23 | 14.116 ± 11.11 | 13.898 ± 11.13 | 13.721 ± 11.04 | 13.315 ± 10.85 | 14.156 ± 11.32 |
| 24 | 12.307 ± 7.22 | 12.377 ± 7.24 | 12.059 ± 7.18 | 11.621 ± 7.04 | 12.23 ± 7.2 |
| 25 | 11.781 ± 7.36 | 11.927 ± 7.4 | 11.601 ± 7.28 | 11.399 ± 7.24 | 11.799 ± 7.39 |
| 26 | 12.107 ± 7.73 | 12.225 ± 7.82 | 11.779 ± 7.7 | 11.551 ± 7.56 | 12.105 ± 7.71 |
| 27 | 13.644 ± 8.19 | 13.79 ± 8.28 | 13.747 ± 8.13 | 13.095 ± 8.06 | 13.373 ± 8.17 |
| 28 | 13.31 ± 8.04 | 13.625 ± 8.14 | 13.118 ± 7.94 | 12.88 ± 7.96 | 13.524 ± 8.13 |
| 29 | 13.247 ± 8.41 | 13.475 ± 8.47 | 13.178 ± 8.36 | 12.741 ± 8.34 | 13.387 ± 8.48 |
| Genome-wide average | 11.89 ± 7.86 | 11.94 ± 7.88 | 11.63 ± 7.8 | 11.27 ± 7.69 | 11.81 ± 7.86 |

*ABR* Abergelle, *AR* Arado, *BG* Begait, *ER* Erob, *RAY* Raya

Table S6. Indel density per kb across each chromosome (mean ± SD).

| Chromosomes | ABR (N = 11) | AR (N = 11) | BG (N = 11) | ER (N = 10) | RAY (N = 11) |
| --- | --- | --- | --- | --- | --- |
| 1 | 1.181 ± 1.37 | 1.186 ± 1.38 | 1.139 ± 1.35 | 1.114 ± 1.34 | 1.182 ± 1.37 |
| 2 | 1.113 ± 1.32 | 1.111 ± 1.32 | 1.08 ± 1.3 | 1.059 ± 1.29 | 1.111 ± 1.32 |
| 3 | 1.067 ± 1.32 | 1.069 ± 1.32 | 1.051 ± 1.31 | 1.024 ± 1.29 | 1.068 ± 1.32 |
| 4 | 1.214 ± 1.45 | 1.216 ± 1.44 | 1.196 ± 1.44 | 1.157 ± 1.41 | 1.207 ± 1.44 |
| 5 | 1.114 ± 1.38 | 1.121 ± 1.38 | 1.087 ± 1.36 | 1.074 ± 1.36 | 1.111 ± 1.38 |
| 6 | 1.187 ± 1.36 | 1.189 ± 1.36 | 1.157 ± 1.35 | 1.115 ± 1.32 | 1.176 ± 1.36 |
| 7 | 1.13 ± 1.39 | 1.145 ± 1.41 | 1.103 ± 1.38 | 1.078 ± 1.36 | 1.132 ± 1.4 |
| 8 | 1.113 ± 1.32 | 1.128 ± 1.33 | 1.096 ± 1.31 | 1.051 ± 1.28 | 1.112 ± 1.32 |
| 9 | 1.152 ± 1.37 | 1.154 ± 1.37 | 1.136 ± 1.35 | 1.104 ± 1.34 | 1.144 ± 1.36 |
| 10 | 1.158 ± 1.43 | 1.159 ± 1.43 | 1.135 ± 1.41 | 1.107 ± 1.4 | 1.155 ± 1.43 |
| 11 | 1.061 ± 1.29 | 1.072 ± 1.3 | 1.062 ± 1.29 | 1.014 ± 1.27 | 1.076 ± 1.31 |
| 12 | 1.294 ± 1.56 | 1.297 ± 1.57 | 1.277 ± 1.55 | 1.233 ± 1.55 | 1.297 ± 1.58 |
| 13 | 1.069 ± 1.32 | 1.081 ± 1.33 | 1.055 ± 1.31 | 1.017 ± 1.28 | 1.075 ± 1.33 |
| 14 | 1.112 ± 1.32 | 1.123 ± 1.33 | 1.08 ± 1.3 | 1.057 ± 1.29 | 1.121 ± 1.33 |
| 15 | 1.295 ± 1.6 | 1.311 ± 1.61 | 1.277 ± 1.6 | 1.253 ± 1.57 | 1.321 ± 1.62 |
| 16 | 1.147 ± 1.38 | 1.145 ± 1.38 | 1.13 ± 1.37 | 1.088 ± 1.34 | 1.153 ± 1.39 |
| 17 | 1.208 ± 1.41 | 1.208 ± 1.41 | 1.177 ± 1.4 | 1.143 ± 1.37 | 1.194 ± 1.4 |
| 18 | 1.116 ± 1.43 | 1.129 ± 1.46 | 1.086 ± 1.43 | 1.073 ± 1.41 | 1.12 ± 1.44 |
| 19 | 1.038 ± 1.3 | 1.053 ± 1.31 | 1.045 ± 1.31 | 0.99 ± 1.26 | 1.069 ± 1.32 |
| 20 | 1.193 ± 1.39 | 1.199 ± 1.4 | 1.135 ± 1.36 | 1.129 ± 1.36 | 1.205 ± 1.4 |
| 21 | 1.16 ± 1.42 | 1.171 ± 1.41 | 1.125 ± 1.4 | 1.115 ± 1.39 | 1.185 ± 1.44 |
| 22 | 1.104 ± 1.35 | 1.102 ± 1.35 | 1.069 ± 1.32 | 1.045 ± 1.31 | 1.111 ± 1.35 |
| 23 | 1.335 ± 1.71 | 1.318 ± 1.71 | 1.306 ± 1.69 | 1.268 ± 1.67 | 1.356 ± 1.76 |
| 24 | 1.183 ± 1.36 | 1.193 ± 1.37 | 1.156 ± 1.35 | 1.121 ± 1.32 | 1.188 ± 1.36 |
| 25 | 1.062 ± 1.33 | 1.078 ± 1.33 | 1.046 ± 1.31 | 1.034 ± 1.3 | 1.077 ± 1.33 |
| 26 | 1.206 ± 1.44 | 1.22 ± 1.46 | 1.167 ± 1.42 | 1.16 ± 1.41 | 1.221 ± 1.45 |
| 27 | 1.338 ± 1.5 | 1.357 ± 1.51 | 1.35 ± 1.5 | 1.291 ± 1.47 | 1.331 ± 1.51 |
| 28 | 1.307 ± 1.51 | 1.344 ± 1.53 | 1.291 ± 1.5 | 1.276 ± 1.49 | 1.345 ± 1.53 |
| 29 | 1.28 ± 1.51 | 1.302 ± 1.54 | 1.276 ± 1.52 | 1.242 ± 1.5 | 1.31 ± 1.54 |
| Genome-wide average | 1.16 ± 1.4 | 1.17 ± 1.41 | 1.14 ± 1.39 | 1.08 ± 1.34 | 1.67 ± 1.41 |

*ABR* Abergelle, *AR* Arado, *BG* Begait, *ER* Erob, *RAY* Raya

Table S7. Distribution of SNPs across each chromosome.

| Chromosomes | ABR (N = 11) | AR (N = 11) | BG (N =11) | ER (N = 10) | RAY (N = 11) |
| --- | --- | --- | --- | --- | --- |
| 1 | 1895194 | 1,900,182 | 1,826,307 | 1,780,125 | 1,880,320 |
| 2 | 1544549 | 1,543,561 | 1,497,209 | 1,462,356 | 1,529,420 |
| 3 | 1339507 | 1,341,661 | 1,317,055 | 1,277,294 | 1,327,538 |
| 4 | 1460244 | 1,458,661 | 1,435,379 | 1,384,129 | 1,434,563 |
| 5 | 1377057 | 1,389,678 | 1,347,155 | 1,322,565 | 1,361,681 |
| 6 | 1411635 | 1,409,455 | 1,372,820 | 1,319,677 | 1,383,440 |
| 7 | 1253734 | 1,266,532 | 1,221,123 | 1,186,635 | 1,243,540 |
| 8 | 1281923 | 1,297,561 | 1,261,815 | 1,201,009 | 1,272,630 |
| 9 | 1217566 | 1,217,681 | 1,198,132 | 1,160,071 | 1,200,497 |
| 10 | 1212396 | 1,214,888 | 1,187,351 | 1,153,221 | 1,200,479 |
| 11 | 1195987 | 1,208,170 | 1,197,704 | 1,141,775 | 1,200,422 |
| 12 | 1143431 | 1,142,167 | 1,122,822 | 1,077,908 | 1,129,950 |
| 13 | 928830 | 939,587 | 916,171 | 877,905 | 924,207 |
| 14 | 949822 | 960,254 | 920,906 | 899,130 | 948,946 |
| 15 | 1112467 | 1,124,249 | 1,093,389 | 1,068,967 | 1,124,157 |
| 16 | 949498 | 948,485 | 932,183 | 894,414 | 943,196 |
| 17 | 892607 | 889,902 | 869,616 | 843,308 | 874,764 |
| 18 | 761353 | 769,041 | 735,312 | 725,745 | 756,596 |
| 19 | 692795 | 702,184 | 696,934 | 658,216 | 708,444 |
| 20 | 879832 | 885,408 | 838,218 | 827,931 | 881,798 |
| 21 | 823442 | 830,629 | 799,752 | 785,910 | 832,481 |
| 22 | 693462 | 688,530 | 666,849 | 650,517 | 685,255 |
| 23 | 741090 | 729,621 | 720,362 | 699,033 | 743,163 |
| 24 | 766788 | 771,143 | 751,405 | 724,076 | 762,019 |
| 25 | 498916 | 505,086 | 491,310 | 482,719 | 499,659 |
| 26 | 629323 | 635,505 | 612,407 | 600,460 | 629,255 |
| 27 | 622327 | 628,981 | 627,025 | 597,296 | 609,951 |
| 28 | 611423 | 625,899 | 602,596 | 591,700 | 621,247 |
| 29 | 676766 | 688,423 | 673,240 | 650,953 | 683,964 |
| Total | 29563964 | 29713124 | 28932547 | 28045045 | 29393582 |

*ABR* Abergelle, *AR* Arado, *BG* Begait, *ER* Erob, *RAY* Raya

Table S8. Distribution of indels across each chromosome.

| Chromosomes | ABR (N = 11) | AR (N = 11) | BG (N = 11) | ER (N = 10) | RAY (N = 11) |
| --- | --- | --- | --- | --- | --- |
| 1 | 187,247 | 188,006 | 180,517 | 176,599 | 187,280 |
| 2 | 151,585 | 151,320 | 146,978 | 144,195 | 151,243 |
| 3 | 129,160 | 129,320 | 127,148 | 123,890 | 129,175 |
| 4 | 145,565 | 145,732 | 143,373 | 138,675 | 144,690 |
| 5 | 133,749 | 134,632 | 130,501 | 129,001 | 133,333 |
| 6 | 139,792 | 140,092 | 136,332 | 131,397 | 138,491 |
| 7 | 125,024 | 126,720 | 122,112 | 119,288 | 125,328 |
| 8 | 126,026 | 127,780 | 124,145 | 118,993 | 125,998 |
| 9 | 120,568 | 120,770 | 118,829 | 115,544 | 119,682 |
| 10 | 119,605 | 119,727 | 117,175 | 114,320 | 119,239 |
| 11 | 113,471 | 114,679 | 113,578 | 108,512 | 115,122 |
| 12 | 112,838 | 113,054 | 111,382 | 107,520 | 113,055 |
| 13 | 89,186 | 90,229 | 88,045 | 84,855 | 89,717 |
| 14 | 91,617 | 92,560 | 88,962 | 87,046 | 92,378 |
| 15 | 110,041 | 111,382 | 108,471 | 106,412 | 112,243 |
| 16 | 92,867 | 92,750 | 91,490 | 88,087 | 93,376 |
| 17 | 88,394 | 88,354 | 86,132 | 83,632 | 87,312 |
| 18 | 73,419 | 74,316 | 71,456 | 70,648 | 73,692 |
| 19 | 65,853 | 66,752 | 66,281 | 62,797 | 67,812 |
| 20 | 85,829 | 86,273 | 81,684 | 81,258 | 86,709 |
| 21 | 81,012 | 81,755 | 78,609 | 77,879 | 82,783 |
| 22 | 67,102 | 66,937 | 64,924 | 63,518 | 67,500 |
| 23 | 70,105 | 69,180 | 68,548 | 66,555 | 71,189 |
| 24 | 73,715 | 74,306 | 72,033 | 69,805 | 74,003 |
| 25 | 44,992 | 45,663 | 44,302 | 43,803 | 45,618 |
| 26 | 62,691 | 63,408 | 60,686 | 60,322 | 63,471 |
| 27 | 61,026 | 61,888 | 61,597 | 58,882 | 60,726 |
| 28 | 60,056 | 61,739 | 59,322 | 58,627 | 61,767 |
| 29 | 65,370 | 66,533 | 65,187 | 63,436 | 66,912 |
| Total | 2,887,905 | 2,905,857 | 2,829,799 | 2,755,496 | 2,899,844 |

*ABR* Abergelle, *AR* Arado, *BG* Begait, *ER* Erob, *RAY* Raya

Table S9. Proportion of alternative allele frequency (AAF) for SNPs and indels with across the Tigray cattle.

| Allelic frequency of detected variants | Population | | | | |
| --- | --- | --- | --- | --- | --- |
| SNPs | Abergelle (N = 11) | Arado (N = 11) | Begait (N = 11) | Erob (N = 10) | Raya (N = 11) |
| No of SNPs with mean AAF < 0.5 (%) | 23430191 (79.25) | 23667911 (79.65) | 22728344 (78.56) | 21625611 (77.11) | 23088375 (78.55) |
| No of SNPs with mean AAF > 0.5 (%) | 6133773 (20.75) | 6045213 (20.35) | 6204203 (21.44) | 6419434 (22.89) | 6305207 (21.45) |
| No of SNPs with mean AAF > 0.9 (%) | 1265652 (4.28) | 1196058 (4.03) | 1323911 (4.58) | 1149665 (4.1) | 1332822 (4.53) |
| µAAF | 0.3 | 0.29 | 0.3 | 0.32 | 0.3 |
| Indels |  |  |  |  |  |
| No of SNPs with mean AAF < 0.5 (%) | 2266334 (79.95) | 2290912 (80.32) | 2200959 (79.27) | 2111465 (78.11) | 2261134 (79.49) |
| No of SNPs with mean AAF > 0.5 (%) | 568422 (20.05) | 561197 (19.68) | 575401 (20.73) | 591902 (21.89) | 583450 (20.51) |
| No of SNPs with mean AAF > 0.9 (%) | 118404 (4.18) | 112639 (3.95) | 123254 (4.44) | 109987 (4.07) | 123839 (4.35) |
| µAAF | 0.29 | 0.28 | 0.29 | 0.3 | 0.28 |

*µAAF* average alternate allele frequency

Table S10. Numerical distribution of nucleotide substitution for the Tigray cattle.

| Nucleotide substitution1 | Population | | | | |
| --- | --- | --- | --- | --- | --- |
|  | ABR (N = 11) | AR (N = 11) | BG (N = 11) | ER (N = 10) | RAY (N = 11) |
| A > C | 1163650 | 1168200 | 1139306 | 1108539 | 1159447 |
| A > G | 4617218 | 4636061 | 4534566 | 4418513 | 4595475 |
| A > T | 879988 | 884155 | 860290 | 835000 | 876229 |
| C > A | 1230556 | 1237089 | 1202191 | 1164066 | 1224922 |
| C > G | 1135067 | 1141536 | 1110983 | 1075914 | 1129324 |
| C > T | 5744637 | 5777051 | 5608171 | 5411407 | 5700535 |
| G > A | 5767713 | 5800307 | 5629465 | 5431403 | 5724353 |
| G > C | 1137839 | 1144012 | 1114739 | 1079326 | 1131548 |
| G > T | 1233745 | 1241488 | 1206275 | 1167903 | 1229929 |
| T > A | 884513 | 889825 | 865341 | 839132 | 881259 |
| T > C | 4607940 | 4626324 | 4523999 | 4408324 | 4584911 |
| T > G | 1161098 | 1167076 | 1137221 | 1105518 | 1155650 |
| TS/TV | 2.349 | 2.349 | 2.35 | 2.349 | 2.345 |

*ABR* Abergelle, *AR* Arado, *BG* Begait, *ER* Erob, *RAY* Raya

Table S11. Distribution of insertions and deletions across the Tigray cattle.

| Indel length in bp | ABR | AR | BG | ER | RAY |
| --- | --- | --- | --- | --- | --- |
| 1 | 1451722 | 1460409 | 1422733 | 1386758 | 1455869 |
| 2 | 455489 | 458417 | 446212 | 435726 | 459339 |
| 3 | 256672 | 257672 | 250611 | 244393 | 257419 |
| 4 | 201403 | 202424 | 196866 | 190997 | 201212 |
| 5 | 104354 | 104799 | 102026 | 98806 | 104213 |
| ≥ 6 | 418265 | 422136 | 411351 | 398816 | 421792 |
| 1bp (%) | 51.21 | 51.2 | 51.24 | 51.3 | 51.18 |
| $\leq$5bp (%) | 87.12 | 87.08 | 87.11 | 87.18 | 87.11 |
| ≥ 6bp (%) | 14.75 | 14.80 | 14.82 | 14.75 | 14.83 |
| Maximum insertion | 20 | 20 | 23 | 21 | 21 |
| Maximum deletion | -28 | -27 | -27 | -27 | -27 |
| Insertion | 1655574 | 1666501 | 1618905 | 1572964 | 1663890 |
| Deletion | 1232331 | 1239356 | 1210894 | 1182532 | 1235954 |
| Insertion/Deletion | 1.34 | 1.34 | 1.34 | 1.33 | 1.35 |

*ABR* Abergelle, *AR* Arado, *BG* Begait, *ER* Erob, *RAY* Raya

Table S12. Description of genome-wide average nucleotide diversity, heterozygous-to-homozygous (Het/Hom) ratios for SNPs and indels, and observed heterozygosity for the Tigray cattle.

| Population | Average nucleotide diversity π | | Het/Hom^1^ | | H_O_^2^ | |
| --- | --- | --- | --- | --- | --- | --- |
|  | Mean | SD | SNPs | Indels | Mean | SD |
| Abergelle | 0.00356 | 0.00176 | 1.64 | 1.61 | 0.287 | 0.010 |
| Arado | 0.00357 | 0.00176 | 1.68 | 1.65 | 0.302 | 0.010 |
| Begait | 0.00351 | 0.00175 | 1.59 | 1.57 | 0.283 | 0.012 |
| Erob | 0.00356 | 0.00177 | 1.60 | 1.58 | 0.284 | 0.008 |
| Raya | 0.00350 | 0.00177 | 1.52 | 1.52 | 0.278 | 0.016 |

^1^Population level average non-reference allele heterozygous to the homozygous ratio

^2^population level average observed heterozygosity

Table S13. ROH statistics in individual samples of the Tigray cattle populations.

| Sample^1^ | Sex | Population | NROH^2^ | Total ROH length in kbp | Average ROH segment length kbp | Minimum ROH length kbp | Maxim ROH length kbp | SNPs |
| --- | --- | --- | --- | --- | --- | --- | --- | --- |
| ABR02 | F | Abergelle | 828 | 128342 | 155.002 | 100.037 | 511.969 | 1855813 |
| ABR07 | F | Abergelle | 843 | 133654 | 158.545 | 100.076 | 743.581 | 1966156 |
| ABR10 | M | Abergelle | 827 | 129313 | 156.364 | 100.004 | 792.798 | 1884691 |
| ABR11 | M | Abergelle | 781 | 126326 | 161.748 | 100.035 | 896.19 | 1853192 |
| ABR12 | F | Abergelle | 748 | 112939 | 150.988 | 100.058 | 547.677 | 1661218 |
| ABR13 | M | Abergelle | 778 | 119221 | 153.24 | 100.002 | 569.855 | 1735227 |
| ABR14 | F | Abergelle | 907 | 140996 | 155.453 | 100.007 | 526.116 | 2076081 |
| ABR15 | F | Abergelle | 900 | 148830 | 165.366 | 100.049 | 787.31 | 2290024 |
| ABR16 | M | Abergelle | 881 | 136537 | 154.98 | 100.131 | 649.346 | 1970678 |
| ABR21 | M | Abergelle | 824 | 131304 | 159.35 | 100.072 | 582.281 | 1920166 |
| ABR29 | F | Abergelle | 814 | 123965 | 152.292 | 100.01 | 608.765 | 1805870 |
| AR01 | F | Arado | 782 | 127175 | 162.628 | 100.152 | 578.938 | 1655364 |
| AR05 | F | Arado | 750 | 118918 | 158.557 | 100.003 | 619.765 | 1718422 |
| AR06 | F | Arado | 835 | 131731 | 157.762 | 100.004 | 605.848 | 1948784 |
| AR10 | M | Arado | 708 | 112441 | 158.815 | 100.003 | 535.183 | 1660465 |
| AR11 | M | Arado | 861 | 135646 | 157.545 | 100.086 | 653.31 | 2011562 |
| AR13 | F | Arado | 711 | 108019 | 151.925 | 100.004 | 572.658 | 1536829 |
| AR23 | F | Arado | 778 | 120078 | 154.342 | 100.047 | 656.572 | 1731512 |
| AR24 | F | Arado | 794 | 124269 | 156.511 | 100.06 | 686.549 | 1816894 |
| AR30 | F | Arado | 838 | 132648 | 158.291 | 100.014 | 523.141 | 1941223 |
| AR31 | F | Arado | 706 | 108766 | 154.06 | 100.028 | 696.998 | 1611131 |
| AR32 | M | Arado | 793 | 122456 | 154.421 | 100.04 | 879.142 | 1789959 |
| BG01 | F | Begait | 908 | 147052 | 161.951 | 100.14 | 660.291 | 2189614 |
| BG02 | F | Begait | 882 | 140682 | 159.504 | 100.11 | 856.199 | 2066527 |
| BG05 | F | Begait | 767 | 121138 | 157.938 | 100.03 | 863.883 | 1768534 |
| BG07 | F | Begait | 849 | 131777 | 155.214 | 100.044 | 699.933 | 1944990 |
| BG13 | F | Begait | 862 | 142015 | 164.75 | 100.069 | 772.951 | 2091634 |
| BG15 | M | Begait | 1504 | 266709 | 177.333 | 100.04 | 1203.302 | 4057130 |
| BG17 | F | Begait | 896 | 150278 | 167.721 | 100.07 | 825.919 | 2208658 |
| BG19 | F | Begait | 940 | 156980 | 167 | 100.012 | 643.085 | 2341021 |
| BG20 | F | Begait | 806 | 127691 | 158.426 | 100.197 | 595.476 | 1823180 |
| BG21 | F | Begait | 782 | 123606 | 158.064 | 100.116 | 582.676 | 1819288 |
| BG31 | F | Begait | 807 | 130173 | 161.305 | 100.091 | 707.055 | 1857526 |
| ER01 | M | Erob | 830 | 131258 | 158.142 | 100.002 | 642.098 | 1965596 |
| ER04 | F | Erob | 1099 | 177459 | 161.474 | 100.163 | 666.114 | 2684630 |
| ER06 | M | Erob | 789 | 125739 | 159.365 | 100.012 | 884.403 | 1860037 |
| ER07 | M | Erob | 786 | 122677 | 156.077 | 100.041 | 520.163 | 1802205 |
| ER10 | F | Erob | 857 | 138470 | 161.575 | 100.072 | 744.868 | 2067601 |
| ER11 | F | Erob | 817 | 130545 | 159.786 | 100.374 | 827.319 | 1904007 |
| ER13 | F | Erob | 810 | 126565 | 156.254 | 100.033 | 583.095 | 1825282 |
| ER15 | F | Erob | 860 | 133288 | 154.986 | 100.068 | 693.829 | 1959606 |
| ER17 | M | Erob | 796 | 122745 | 154.202 | 100.014 | 608.262 | 1796287 |
| ER18 | F | Erob | 727 | 110403 | 151.861 | 100.134 | 505.474 | 1609470 |
| RAY05 | M | Raya | 867 | 137997 | 159.166 | 100.132 | 917.33 | 2013202 |
| RAY06 | M | Raya | 703 | 106297 | 151.205 | 100.011 | 590.014 | 1546638 |
| RAY11 | F | Raya | 1662 | 297294 | 178.877 | 100.019 | 1180.322 | 4765199 |
| RAY17 | F | Raya | 826 | 131153 | 158.781 | 100.005 | 974.976 | 1949028 |
| RAY19 | F | Raya | 890 | 140242 | 157.575 | 100.048 | 572.079 | 2059272 |
| RAY21 | F | Raya | 811 | 127877 | 157.678 | 100.067 | 724.247 | 1860730 |
| RAY22 | F | Raya | 1467 | 253580 | 172.856 | 100.008 | 1040.337 | 3916061 |
| RAY23 | F | Raya | 710 | 105866 | 149.108 | 100.004 | 604.694 | 1532947 |
| RAY25 | F | Raya | 860 | 139991 | 162.781 | 100.031 | 595.453 | 2069018 |
| RAY26 | F | Raya | 1347 | 220110 | 163.408 | 100.097 | 1002.7 | 3297432 |
| RAY30 | F | Raya | 862 | 133322 | 154.666 | 100.045 | 581.401 | 1913403 |

^1^*ABR** Abergelle, *RA** Arado, *BG** Begait, *ER** Erob, and *RAY** Raya

*NROH^2^* Number runs of homozygosity

Table 14. Abundance of ROH across different ROH segment length categories (0.1 - 0.25 Mbp, > 0.25 - 0.5 Mbp, > 0.5 - 1 Mbp and > 1 Mbp) supplementary.

| Breed^1^ | 0.1 - 0.25Mbp | | > 0.25 - 0.5 Mbp | | > 0.5 - 1Mbp | | > 1Mbp | |
| --- | --- | --- | --- | --- | --- | --- | --- | --- |
|  | Count | % | Count | % | Count | % | Count | % |
| ABR | 8440 | 92.4 | 670 | 7.3 | 21 | 0.2 | 0 | 0 |
| AR | 7875 | 92 | 665 | 7.8 | 16 | 0.2 | 0 | 0 |
| BG | 8964 | 89.6 | 994 | 9.9 | 44 | 0.4 | 1 | 0.01 |
| ER | 7680 | 91.7 | 669 | 8 | 22 | 0.3 | 0 | 0 |
| RAY | 9947 | 90.3 | 1010 | 9.2 | 45 | 0.4 | 3 | 0.04 |
| Eth.Boran | 8094 | 90.6 | 815 | 9.1 | 28 | 0.3 | 0 | 0 |
| KEN | 4204 | 90.9 | 394 | 8.5 | 25 | 0.5 | 1 | 0 |
| FOG | 7118 | 91.1 | 678 | 8.7 | 21 | 0.3 | 0 | 0 |
| HOR | 7850 | 92.3 | 632 | 7.4 | 24 | 0.3 | 2 | 0 |
| ANK | 2249 | 97.8 | 50 | 2.2 | 0 | 0 | 0 | 0 |
| AFR | 6865 | 92.3 | 565 | 7.6 | 11 | 0.1 | 0 | 0 |
| HOL | 17821 | 80.6 | 3828 | 17.3 | 436 | 2 | 39 | 0.2 |
| AAN | 18819 | 81.8 | 3762 | 16.4 | 392 | 1.7 | 26 | 0.1 |
| MUT | 6528 | 96.6 | 223 | 3.3 | 4 | 0.1 | 0 | 0 |
| NDA | 6678 | 97.2 | 192 | 2.8 | 0 | 0 | 0 | 0 |
| As. zebu | 1236 | 95.7 | 56 | 4.3 | 0 | 0 | 0 | 0 |

^1^*ABR* Abergelle, *AR* Arado, *BG* Begait, *ER* Erob, *RAY* Raya, *Eth.Boran* Ethiopian Boran, *KEN* Kenana, *FOG* Fogera, *HO*R Horro, *ANK* Ankole, *AFA* Afar, *HOL* Holstein, *ANG* Angus, *MUT* Mururu, *NDA* Ndama, *As. zebu* Asian zebu

Table 15. Average genomic size of ROH across different length categories (0.1 - 0.25 Mbp, > 0.25 - 0.5 Mbp, > 0.5 - 1 Mbp and > 1 Mbp).

| Breeds | 0.1 - 0.25 Mbp (%) | > 0.25 - 0.5 Mbp (%) | > 0.5 - 1 Mbp (%) | > 1 Mbp (%) |
| --- | --- | --- | --- | --- |
|  | Mbp (%) | Mbp (%) | Mbp (%) | Mbp (%) |
| Abergelle | 109.1 (83.84) | 19.76 (15.19) | 1.26 (1) | 0 (0) |
| Arado | 101.7 (83.35) | 19.4 (15.9) | 0.92 (1) | 0 (0) |
| Begait | 115.9 (77.84) | 29.21 (19.62) | 2.59 (1.74) | 1.2 (0.81) |
| Erob | 109.06 (82.68) | 21.41 (16.23) | 1.44 (1) | 0 (0) |
| Raya | 126.09 (76.94) | 29.78 (18.26) | 4.81 (2) | 3.2 (1.95) |
| Eth.Boran | 115.89 (80.51) | 26.17 (18.18) | 1.89 (1) | 0 (0) |
| Kenana | 59.21 (80.25) | 12.88 (17.46) | 1.59 (2) | 0.1 (0.14) |
| Fogera | 112.54 (81.36) | 24.31 (17.57) | 1.48 (1) | 0 (0) |
| Horro | 101.92 (83.72) | 18.24 (14.98) | 1.38 (1) | 0.2 (0.16) |
| Ankole | 29.63 (95.11) | 1.52 (4.89) | 0 (0) | 0 (0) |
| Afar | 96.48 (83.74) | 18.02 (15.64) | 0.71 (1) | 0 (0) |
| Holstein | 267.28 (61.78) | 130.96 (30.27) | 29.7 (7) | 4.68 (1.08) |
| Angus | 282.03 (64.2) | 127.85 (29.1) | 26.38 (6) | 3.05 (0.69) |
| Muturu | 87.74 (92.48) | 6.9 (7.27) | 0.24 (0) | 0 (0) |
| N’Dama | 88.77 (93.94) | 5.72 (6.06) | 0 (0) | 0 (0) |
| Asian zebu | 18.42 (90.33) | 1.97 (9.67) | 0 (0) | 0 (0) |

Table S16. Chromosome wise number of ROH across the Tigray cattle population.

| CHR | ABR |  | AR |  | | BG |  | ER |  | RAY |  |
| --- | --- | --- | --- | --- | --- | --- | --- | --- | --- | --- | --- |
|  | Sum | AVG | Sum | AVG | Sum | | AVG | Sum | AVG | Sum | AVG |
| 1 | 600 | 55 | 549 | 50 | 527 | | 48 | 635 | 64 | 633 | 58 |
| 2 | 543 | 49 | 569 | 52 | 608 | | 55 | 467 | 47 | 623 | 57 |
| 3 | 445 | 40 | 408 | 37 | 464 | | 42 | 426 | 43 | 492 | 45 |
| 4 | 423 | 38 | 391 | 36 | 348 | | 32 | 346 | 35 | 674 | 61 |
| 5 | 482 | 44 | 452 | 41 | 645 | | 59 | 399 | 40 | 467 | 42 |
| 6 | 441 | 40 | 374 | 34 | 471 | | 43 | 512 | 51 | 627 | 57 |
| 7 | 471 | 43 | 493 | 45 | 508 | | 46 | 449 | 45 | 532 | 48 |
| 8 | 422 | 38 | 368 | 33 | 433 | | 39 | 473 | 47 | 536 | 49 |
| 9 | 390 | 35 | 404 | 37 | 434 | | 39 | 296 | 30 | 575 | 52 |
| 10 | 352 | 32 | 340 | 31 | 433 | | 39 | 356 | 36 | 480 | 44 |
| 11 | 438 | 40 | 387 | 35 | 426 | | 39 | 416 | 42 | 445 | 40 |
| 12 | 282 | 26 | 253 | 23 | 271 | | 25 | 218 | 22 | 412 | 37 |
| 13 | 282 | 26 | 261 | 24 | 371 | | 34 | 289 | 29 | 423 | 38 |
| 14 | 276 | 25 | 259 | 24 | 445 | | 40 | 294 | 29 | 364 | 33 |
| 15 | 267 | 24 | 275 | 25 | 270 | | 25 | 140 | 14 | 354 | 32 |
| 16 | 321 | 29 | 308 | 28 | 406 | | 37 | 289 | 29 | 435 | 40 |
| 17 | 235 | 21 | 250 | 23 | 266 | | 24 | 280 | 28 | 449 | 41 |
| 18 | 242 | 22 | 226 | 21 | 297 | | 27 | 213 | 21 | 227 | 21 |
| 19 | 292 | 27 | 281 | 26 | 265 | | 24 | 228 | 23 | 259 | 24 |
| 20 | 242 | 22 | 237 | 22 | 297 | | 27 | 234 | 23 | 266 | 24 |
| 21 | 260 | 24 | 222 | 20 | 379 | | 34 | 204 | 20 | 236 | 21 |
| 22 | 285 | 26 | 235 | 21 | 279 | | 25 | 245 | 25 | 242 | 22 |
| 23 | 144 | 13 | 135 | 12 | 163 | | 15 | 119 | 12 | 151 | 14 |
| 24 | 221 | 20 | 217 | 20 | 215 | | 20 | 192 | 19 | 201 | 18 |
| 25 | 135 | 12 | 141 | 13 | 193 | | 18 | 109 | 11 | 194 | 18 |
| 26 | 175 | 16 | 124 | 11 | 170 | | 15 | 207 | 21 | 155 | 14 |
| 27 | 144 | 13 | 131 | 12 | 116 | | 11 | 112 | 11 | 196 | 18 |
| 28 | 156 | 14 | 128 | 12 | 153 | | 14 | 114 | 11 | 177 | 16 |
| 29 | 165 | 15 | 138 | 13 | 150 | | 14 | 109 | 11 | 180 | 16 |

*CHR* Chromosome, *AVG* Average, *ABR* Abergelle, *AR* Arado, *BG* Begait, *ER* Erob, *RAY* Raya

Table S17. Chromosome wise average size of ROH (in megabyte) of the Tigray cattle population.

| CHR | ABR |  | AR |  | | BG | |  | ER |  | RAY |  |
| --- | --- | --- | --- | --- | --- | --- | --- | --- | --- | --- | --- | --- |
|  | Sum | AVG | Sum | AVG | | Sum | | AVG | Sum | AVG | Sum | AVG |
| 1 | 93 | 8.45 | 86 | | 7.82 | | 85 | 7.73 | 100 | 10.00 | 104 | 9.45 |
| 2 | 90 | 8.18 | 92 | | 8.36 | | 100 | 9.09 | 76 | 7.60 | 104 | 9.45 |
| 3 | 72 | 6.55 | 63 | | 5.73 | | 73 | 6.64 | 67 | 6.70 | 77 | 7.00 |
| 4 | 64 | 5.82 | 59 | | 5.36 | | 59 | 5.36 | 51 | 5.10 | 110 | 10.00 |
| 5 | 74 | 6.73 | 71 | | 6.45 | | 112 | 10.18 | 61 | 6.10 | 73 | 6.64 |
| 6 | 68 | 6.18 | 58 | | 5.27 | | 73 | 6.64 | 84 | 8.40 | 100 | 9.09 |
| 7 | 73 | 6.64 | 75 | | 6.82 | | 81 | 7.36 | 73 | 7.30 | 84 | 7.64 |
| 8 | 69 | 6.27 | 59 | | 5.36 | | 68 | 6.18 | 78 | 7.80 | 86 | 7.82 |
| 9 | 62 | 5.64 | 65 | | 5.91 | | 73 | 6.64 | 48 | 4.80 | 99 | 9.00 |
| 10 | 55 | 5.00 | 52 | | 4.73 | | 71 | 6.45 | 56 | 5.60 | 83 | 7.55 |
| 11 | 70 | 6.36 | 67 | | 6.09 | | 70 | 6.36 | 64 | 6.40 | 70 | 6.36 |
| 12 | 43 | 3.91 | 38 | | 3.45 | | 43 | 3.91 | 32 | 3.20 | 68 | 6.18 |
| 13 | 44 | 4.00 | 40 | | 3.64 | | 63 | 5.73 | 46 | 4.60 | 69 | 6.27 |
| 14 | 43 | 3.91 | 40 | | 3.64 | | 73 | 6.64 | 45 | 4.50 | 60 | 5.45 |
| 15 | 39 | 3.55 | 43 | | 3.91 | | 41 | 3.73 | 21 | 2.10 | 56 | 5.09 |
| 16 | 50 | 4.55 | 47 | | 4.27 | | 69 | 6.27 | 46 | 4.60 | 71 | 6.45 |
| 17 | 38 | 3.45 | 38 | | 3.45 | | 41 | 3.73 | 45 | 4.50 | 84 | 7.64 |
| 18 | 39 | 3.55 | 37 | | 3.36 | | 52 | 4.73 | 34 | 3.40 | 38 | 3.45 |
| 19 | 45 | 4.09 | 45 | | 4.09 | | 43 | 3.91 | 36 | 3.60 | 42 | 3.82 |
| 20 | 37 | 3.36 | 38 | | 3.45 | | 48 | 4.36 | 36 | 3.60 | 42 | 3.82 |
| 21 | 40 | 3.64 | 36 | | 3.27 | | 68 | 6.18 | 32 | 3.20 | 37 | 3.36 |
| 22 | 45 | 4.09 | 37 | | 3.36 | | 46 | 4.18 | 39 | 3.90 | 38 | 3.45 |
| 23 | 22 | 2.00 | 19 | | 1.73 | | 24 | 2.18 | 18 | 1.80 | 22 | 2.00 |
| 24 | 34 | 3.09 | 35 | | 3.18 | | 34 | 3.09 | 29 | 2.90 | 32 | 2.91 |
| 25 | 19 | 1.73 | 21 | | 1.91 | | 33 | 3.00 | 16 | 1.60 | 30 | 2.73 |
| 26 | 28 | 2.55 | 20 | | 1.82 | | 27 | 2.45 | 33 | 3.30 | 24 | 2.18 |
| 27 | 22 | 2.00 | 21 | | 1.91 | | 18 | 1.64 | 17 | 1.70 | 34 | 3.09 |
| 28 | 26 | 2.36 | 19 | | 1.73 | | 22 | 2.00 | 17 | 1.70 | 29 | 2.64 |
| 29 | 25 | 2.27 | 21 | | 1.91 | | 24 | 2.18 | 17 | 1.70 | 27 | 2.45 |

*CHR* Chromosome*, AVG* Average, *ABR* Abergelle, *AR* Arado, *BG* Begait, *ER* Erob, *RAY* Raya

Table S18. Chromosome wise incidence of SNPs on ROH across the Tigray cattle population.

| CHR | ABR | AR | BG | ER | RAY |
| --- | --- | --- | --- | --- | --- |
|  | Sum | Sum | Sum | Sum | Sum |
| 1 | 127885 | 113383 | 116370 | 157209 | 143208 |
| 2 | 117423 | 118571 | 129131 | 109155 | 140474 |
| 3 | 92055 | 81256 | 94454 | 96197 | 101379 |
| 4 | 83466 | 77508 | 77727 | 72729 | 146011 |
| 5 | 93217 | 90461 | 144672 | 84575 | 93041 |
| 6 | 94542 | 82759 | 104415 | 131581 | 144961 |
| 7 | 85267 | 91627 | 99192 | 96636 | 104169 |
| 8 | 89971 | 77752 | 89882 | 112888 | 117202 |
| 9 | 82661 | 86825 | 97239 | 72362 | 134144 |
| 10 | 70247 | 66233 | 92406 | 77899 | 111736 |
| 11 | 94686 | 87331 | 93446 | 93163 | 92819 |
| 12 | 60274 | 51498 | 62334 | 50943 | 101137 |
| 13 | 52584 | 47817 | 81102 | 63335 | 87158 |
| 14 | 57570 | 53888 | 100450 | 67694 | 84051 |
| 15 | 56649 | 64325 | 60034 | 34088 | 82896 |
| 16 | 67522 | 62812 | 93698 | 69512 | 96626 |
| 17 | 52136 | 52862 | 56440 | 66974 | 117370 |
| 18 | 48353 | 46217 | 66764 | 47426 | 46813 |
| 19 | 57869 | 54964 | 53338 | 47677 | 51277 |
| 20 | 52695 | 53823 | 67977 | 55276 | 59750 |
| 21 | 54841 | 47417 | 88838 | 47286 | 49170 |
| 22 | 55774 | 46548 | 57371 | 53716 | 47106 |
| 23 | 32572 | 30685 | 35478 | 28654 | 33665 |
| 24 | 48517 | 48467 | 47604 | 46180 | 44590 |
| 25 | 27160 | 29512 | 45730 | 25492 | 43660 |
| 26 | 40574 | 27365 | 36599 | 51910 | 33127 |
| 27 | 33811 | 31288 | 28069 | 29191 | 52670 |
| 28 | 41402 | 31482 | 37730 | 29073 | 46658 |
| 29 | 39102 | 32456 | 38610 | 28652 | 40671 |
| Sum | 1910825 | 1787132 | 2197100 | 1947473 | 2447539 |

*CHR* Chromosome*, ABR* Abergelle, *AR* Arado, *BG* Begait, *ER* Erob, *RAY* Raya

Table S19. Population level mean genomic inbreeding coefficients calculated from the excess of homozygosity (F_HOM_) and from ROH with a minimum length of 100 Kb (F_ROH_ *>*100).

| Breeds | F_HOM_ | | F_ROH_ | |
| --- | --- | --- | --- | --- |
|  | Mean | SD | Mean | SD |
| Abergelle | 0.045 | 0.028 | 0.050 | 0.004 |
| Arado | 0.043 | 0.035 | 0.047 | 0.004 |
| Begait | 0.059 | 0.040 | 0.058 | 0.016 |
| Erob | 0.046 | 0.030 | 0.051 | 0.007 |
| Raya | 0.070 | 0.054 | 0.064 | 0.025 |

Table S20. F_HOM_ and F_ROH_ based inbreeding for the individual animal within each of the Tigray cattle populations.

| Sample^1^ | Sex | Population | Inbreeding coefficient | |
| --- | --- | --- | --- | --- |
|  |  |  | F_HOM_ | F_ROH_ |
| ABR02 | F | Abergelle | 0.041 | 0.050 |
| ABR07 | F | Abergelle | 0.060 | 0.053 |
| ABR10 | M | Abergelle | 0.076 | 0.050 |
| ABR11 | M | Abergelle | 0.004 | 0.049 |
| ABR12 | F | Abergelle | 0.074 | 0.044 |
| ABR13 | M | Abergelle | 0.011 | 0.046 |
| ABR14 | F | Abergelle | 0.021 | 0.054 |
| ABR15 | F | Abergelle | 0.016 | 0.057 |
| ABR16 | M | Abergelle | 0.075 | 0.053 |
| ABR21 | M | Abergelle | 0.054 | 0.051 |
| ABR29 | F | Abergelle | 0.069 | 0.048 |
| AR01 | F | Arado | 0.002 | 0.048 |
| AR05 | F | Arado | 0.026 | 0.046 |
| AR06 | F | Arado | 0.072 | 0.051 |
| AR10 | M | Arado | 0.057 | 0.043 |
| AR11 | M | Arado | 0.050 | 0.052 |
| AR13 | F | Arado | 0.035 | 0.041 |
| AR23 | F | Arado | 0.017 | 0.046 |
| AR24 | F | Arado | 0.007 | 0.048 |
| AR30 | F | Arado | 0.015 | 0.051 |
| AR31 | F | Arado | 0.115 | 0.042 |
| AR32 | M | Arado | 0.074 | 0.048 |
| BG01 | F | Begait | 0.038 | 0.057 |
| BG02 | F | Begait | 0.065 | 0.054 |
| BG05 | F | Begait | -0.016 | 0.047 |
| BG07 | F | Begait | 0.091 | 0.051 |
| BG13 | F | Begait | 0.069 | 0.055 |
| BG15 | M | Begait | 0.107 | 0.103 |
| BG17 | F | Begait | 0.017 | 0.058 |
| BG19 | F | Begait | 0.006 | 0.060 |
| BG20 | F | Begait | 0.072 | 0.050 |
| BG21 | F | Begait | 0.046 | 0.048 |
| BG31 | F | Begait | -0.005 | 0.050 |
| ER01 | M | Erob | 0.031 | 0.050 |
| ER04 | F | Erob | -0.001 | 0.070 |
| ER06 | M | Erob | 0.091 | 0.048 |
| ER07 | M | Erob | 0.085 | 0.048 |
| ER10 | F | Erob | 0.069 | 0.054 |
| ER11 | F | Erob | 0.079 | 0.051 |
| ER13 | F | Erob | 0.074 | 0.049 |
| ER15 | F | Erob | 0.057 | 0.051 |
| ER17 | M | Erob | 0.026 | 0.049 |
| ER18 | F | Erob | 0.081 | 0.043 |
| RAY05 | M | Raya | 0.094 | 0.053 |
| RAY06 | M | Raya | 0.026 | 0.042 |
| RAY11 | F | Raya | 0.120 | 0.114 |
| RAY17 | F | Raya | 0.016 | 0.051 |
| RAY19 | F | Raya | 0.056 | 0.054 |
| RAY21 | F | Raya | 0.066 | 0.050 |
| RAY22 | F | Raya | 0.168 | 0.099 |
| RAY23 | F | Raya | 0.013 | 0.053 |
| RAY25 | F | Raya | -0.001 | 0.053 |
| RAY26 | F | Raya | 0.111 | 0.088 |
| RAY30 | F | Raya | 0.106 | 0.042 |

^1^*ABR** Abergelle, *RA** Arado, *BG** Begait, *ER** Erob, and *RAY** Raya

Table S21. Population differentiation (*F_ST_*) among the Tigray cattle population and with representative groups of cattle populations

|  | ABR | AR | BG | ER | RAY | Eth.Boran | KEN | FOG | HOR | ANK | AFA | HOIL | ANG | MUT | NDA | As. zebu |
| --- | --- | --- | --- | --- | --- | --- | --- | --- | --- | --- | --- | --- | --- | --- | --- | --- |
| ABR | 0.000 | 0.001 | 0.016 | 0.014 | 0.003 | 0.010 | 0.025 | 0.006 | 0.007 | 0.086 | 0.012 | 0.307 | 0.309 | 0.333 | 0.241 | 0.070 |
| AR |  | 0.000 | 0.015 | 0.015 | 0.002 | 0.009 | 0.024 | 0.006 | 0.007 | 0.084 | 0.010 | 0.300 | 0.303 | 0.328 | 0.236 | 0.070 |
| BG |  |  | 0.000 | 0.026 | 0.018 | 0.025 | 0.023 | 0.022 | 0.025 | 0.101 | 0.026 | 0.312 | 0.315 | 0.338 | 0.246 | 0.077 |
| ER |  |  |  | 0.000 | 0.017 | 0.024 | 0.037 | 0.022 | 0.023 | 0.108 | 0.026 | 0.332 | 0.335 | 0.360 | 0.264 | 0.080 |
| RAY |  |  |  |  | 0.000 | 0.010 | 0.029 | 0.009 | 0.011 | 0.092 | 0.010 | 0.314 | 0.317 | 0.340 | 0.248 | 0.070 |
| Eth.Boran |  |  |  |  |  | 0.000 | 0.030 | 0.011 | 0.011 | 0.091 | 0.013 | 0.322 | 0.325 | 0.352 | 0.255 | 0.074 |
| KEN |  |  |  |  |  |  | 0.000 | 0.023 | 0.023 | 0.087 | 0.031 | 0.304 | 0.307 | 0.335 | 0.233 | 0.084 |
| FOG |  |  |  |  |  |  |  | 0.000 | 0.003 | 0.079 | 0.013 | 0.319 | 0.322 | 0.349 | 0.246 | 0.076 |
| HOR |  |  |  |  |  |  |  |  | 0.000 | 0.069 | 0.014 | 0.290 | 0.292 | 0.315 | 0.221 | 0.078 |
| ANK |  |  |  |  |  |  |  |  |  | 0.000 | 0.096 | 0.253 | 0.255 | 0.276 | 0.183 | 0.178 |
| AFA |  |  |  |  |  |  |  |  |  |  | 0.000 | 0.326 | 0.328 | 0.355 | 0.257 | 0.074 |
| HOL |  |  |  |  |  |  |  |  |  |  |  | 0.000 | 0.125 | 0.312 | 0.259 | 0.393 |
| ANG |  |  |  |  |  |  |  |  |  |  |  |  | 0.000 | 0.320 | 0.261 | 0.396 |
| MUT |  |  |  |  |  |  |  |  |  |  |  |  |  | 0.000 | 0.235 | 0.435 |
| NDA |  |  |  |  |  |  |  |  |  |  |  |  |  |  | 0.000 | 0.338 |
| As. zebu |  |  |  |  |  |  |  |  |  |  |  |  |  |  |  | 0.000 |

^1^*ABR* Abergelle, *AR* Arado, *BG* Begait, *ER* Erob, *RAY* Raya, *Eth.Boran* Ethiopian Boran, *KEN* Kenana, *FOG* Fogera, *HO*R Horro, *ANK* Ankole, *AFA* Afar, *HOL* Holstein, *ANG* Angus, *MUT* Mururu, *NDA* Ndama, *As. zebu* Asian zebu.
